# Dynamics of the Transcriptome and Accessible Chromatin Landscapes During Early Goose Ovarian Development

**DOI:** 10.3389/fcell.2020.00196

**Published:** 2020-04-03

**Authors:** Shenqiang Hu, Shuang Yang, Yao Lu, Yan Deng, Li Li, Jiaran Zhu, Yuan Zhang, Bo Hu, Jiwei Hu, Lu Xia, Hua He, Chunchun Han, Hehe Liu, Bo Kang, Liang Li, Jiwen Wang

**Affiliations:** Farm Animal Genetic Resources Exploration and Innovation Key Laboratory of Sichuan Province, Sichuan Agricultural University, Chengdu, China

**Keywords:** ovary, peri-hatching oocyte loss, primordial follicle formation and development, chromatin accessibility, ATAC-seq, transcriptome sequencing

## Abstract

In contrast to the situation in mammals, very little is known about the molecular mechanisms regulating early avian ovarian development. This study aimed to investigate the dynamic changes in the histomorphology as well as the genome-wide transcriptome and chromatin accessibility landscapes of the goose ovary during late embryonic and early post-hatching stages. Results from hematoxylin-eosin, periodic acid-Schiff, and anti-CVH immunohistochemical stainings demonstrated that programmed oocyte loss, oocyte nest breakdown and primordial follicle formation, and the primordial-to-secondary follicle transition occur during the periods from embryonic day 15 (E15) to post-hatching day 0 (P0), from P0 to P4, and from P4 to P28, respectively. RNA-seq and ATAC-seq analyses revealed dynamic changes in both the ovarian transcriptome and accessible chromatin landscapes during early ovarian development, exhibiting the most extensive changes during peri-hatching oocyte loss, and moreover, differences were also identified in the genomic distribution of the differential ATAC-seq peaks between different developmental stages, suggesting that chromatin-level regulation of gene expression is facilitated by modulating the accessibility of different functional genomic regions to transcription factors. Motif analysis of developmental stage-selective peak regions identified hundreds of potential *cis*-regulatory elements that contain binding sites for many transcription factors, including SF1, NR5A2, ESRRβ, NF1, and THRβ, as well as members of the GATA, SMAD, and LHX families, whose expression fluctuated throughout early goose ovarian development. Integrated ATAC-seq and RNA-seq analysis suggested that the number and genomic distribution of the newly appeared and disappeared peaks differed according to developmental stage, and in combination with qRT-PCR validation potentiated the critical actions of the DEGs enriched in cell cycle, MAPK signaling, and FoxO signaling pathways during peri-hatching oocyte loss and those in ligand–receptor interaction, tissue remodeling, lipid metabolism, and Wnt signaling during primordial follicle formation and development. In conclusion, our study provides a framework for understanding the transcriptome and accessible chromatin dynamics during early avian ovarian development and a new avenue to unravel the transcriptional regulatory mechanisms that facilitate the occurrence of relevant molecular events.

## Introduction

Fertility in female vertebrates is determined by the total number of oocytes destined for ovulation during their entire reproductive life, which depends on the size of the ovarian reserve as well as the developmental process, termed folliculogenesis (Broekmans et al., 2007). A pool of primordial follicles, which consist of a quiescent oocyte in the diplotene stage of meiosis prophase I surrounded by a layer of flattened granulosa cells and assembled perinatally, constitute the ovarian reserve that is widely accepted to represent the only lifetime oocyte source of a female, and it is noteworthy that germline cyst breakdown and programmed oocyte loss occurring during late fetal and early neonatal life drastically decrease the size of the ovarian reserve in a wide variety of species (Pepling, 2006; Hunt and Hassold, 2008; Monget et al., 2012). Regarding the fate of the resting primordial follicles, the majority is presumed to undergo apoptosis during lifespan and only a few are ultimately ovulated through normal folliculogenesis (Monget et al., 2012). Folliculogenesis starts with the recruitment of primordial follicles into the growth phase to become primary follicles and then develops through their transition to preantral, antral, and preovulatory follicles, and culminates with the production of a mature follicle able to ovulate a fertilizable oocyte (Tingen et al., 2009). Decreased ovarian reserve and aberrant folliculogenesis due to intrinsic and/or extrinsic factors would cause not only infertility in mammals but also impaired fecundity in birds (Levi et al., 2001; Krysko et al., 2008; Johnson, 2011), and therefore, it is of great significance to elucidate the mechanisms controlling oocyte growth and follicle development in both species.

Given the large size and easy accessibility of antral follicles, most studies have been focused on the later stages of follicle development (Fortune et al., 2000). By comparison, much less is known about the dynamics and mechanisms of early follicle development. Over the last 20 years, with the urge demand for solving the infertility problems related to early ovarian development, the improved experimental systems, and the advent of high-throughput sequencing technologies, considerable progresses have been made in understanding the critical molecular events during early follicle development, including perinatal oocyte loss, primordial follicle formation, and the primordial-to-secondary follicle transition, as well as the underlying molecular mechanisms in a range of mammalian species (Wang et al., 2017). One of the most exciting progresses in the first decade is the illustration of the pivotal roles of intra-ovarian factors (especially oocyte-derived ones) and oocyte–somatic cell interaction during primordial follicle formation and development (Guigon and Magre, 2006; Hsueh et al., 2015), and these events have recently been demonstrated to be associated with dynamic reorganization of open chromatin in both oocytes and somatic cells (Apostolou and Hochedlinger, 2013; Miyamoto et al., 2018; Gu et al., 2019). In contrast, there is almost a paucity of information about the accessible chromatin dynamic profiles during ovarian development in birds, although some epigenetic changes, such as chromatin remodeling, cytosine methylation, histone modification, and non-coding RNAs, have been evidenced to regulate avian ovarian cell functions (Krasikova et al., 2012; Guioli and Lovell-Badge, 2016; Li et al., 2019). At the transcriptomic level, a growing body of literature is emerging regarding genome-wide gene expression differences between ovaries of different breeds, or ovaries at different physiological stages, or ovarian follicles of different size class, or different types of ovarian cells, which assist in identification of a number of factors regulating avian ovarian functions (Kang et al., 2013; Xu et al., 2013; Hu et al., 2014; Li et al., 2019). Nevertheless, in addition to functional studies of a few genes (Johnson and Woods, 2009; Guioli et al., 2014; Zhao et al., 2017), very little is known about the critical events and regulation of early avian follicle development, including peri-hatching oocyte loss, oocyte nest breakdown and primordial follicle formation, and the primordial-to-secondary follicle transition.

To address these knowledge gaps on early avian ovarian development, in the present study, histomorphological and immunohistochemical characterization of the abovementioned molecular events was firstly carried out in ovaries of embryonic and early post-hatching geese (*Anser cygnoides*). Then, RNA-seq was employed to reveal differences in global gene expression patterns between ovaries at four representative stages of development. Also, we established the dynamic genome-wide chromatin accessibility landscapes by detecting regions of open chromatin using the Assay for Transposase-Accessible Chromatin with high-throughput sequencing (ATAC-seq) (Buenrostro et al., 2013). Finally, integrative analysis of RNA-seq and ATAC-seq data was performed to reveal the correlation of chromatin accessibility with dynamic transcriptomic changes and to identify crucial pathways and genes involved in early ovarian development. These results are expected to shed new light on the molecular mechanisms regulating early ovarian development in birds.

## Materials and Methods

### Ethics Statement

All experimental procedures involving the manipulation of birds were conducted in concordance with the “Guidelines for Experimental Animals” of the Ministry of Science and Technology (Beijing, China). This study was reviewed and approved by the Institutional Animal Care and Use Committee (IACUC) of Sichuan Agricultural University (Chengdu campus, Sichuan, China).

### Experimental Birds and Tissue Collection

Female Tianfu Meat geese (*Anser cygnoides*, laying an average number of 70–90 eggs per year), hatched from the same batch of fertilized eggs obtained at the Waterfowl Breeding Experimental Farm of Sichuan Agricultural University (Ya’an campus, Sichuan, China), during embryonic and early post-hatching periods were used in this study. Embryonic brain/heart tissue was used for sex identification via PCR amplification of the chromodomain helicase DNA binding protein 1 (*CHD1*) gene sequence using this primer set (F: 5′-TGCAGAAGCAATATTACAAGT-3′; R: 5′-AATTCATTATCATCTGGTGG-3′), as previously described in Liu et al. (2014). Thereafter, the left gonads were collected on the embryonic day 6 (E6), E12, E15, and E26, as well as on the post-hatching day 0 (P0), P4, P7, P14, P21, and P28, respectively.

### Histological and Immunohistochemical Observation

The left gonads were 4% formaldehyde-fixed for 72 h at room temperature, dehydrated through a graded ethanol series, transferred to xylene, and embedded in paraffin-wax. Paraffin sections of 5 μm thickness from each gonad were stained with hematoxylin-eosin (HE) and photographed under a Nikon 90i microscope (Nikon, Japan). Besides, the sections from E15 and E26 gonads were also subjected to periodic acid-Schiff (PAS) and immunohistochemical stainings. For immunohistochemistry, after dewaxing and hydration, the left gonad sections were incubated in 3% H_2_O_2_ in the dark for 25 min to block endogenous peroxidase and then washed three times with phosphate-buffered saline (PBS) for 5 min each. Thereafter, the sections were incubated with blocking buffer (ZSGB-BIO, Beijing, China) at room temperature for 30 min, followed by the rabbit-anti-chicken vasa homolog (CVH)/DEAD box polypeptide 4 (DDX4) primary antibody (Bioss Antibodies, Woburn, MA, United States) at 4°C overnight. After being washed three times with PBS for 5 min each, the sections were incubated with the horseradish peroxidase (HRP)-conjugated goat-anti-rabbit IgG secondary antibody (ZSGB-BIO, Beijing, China) at 37°C for 30 min. Finally, the sections were exposed to a diaminobenzidine (DAB) solution (ZSGB-BIO, Beijing, China), rinsed in distilled water, counterstained with hematoxylin, and photographed under a Nikon 90i microscope (Nikon, Japan).

### RNA-Seq Library Preparation, Sequencing, and Analysis

The left gonads of females at four representative stages of development (i.e., E15, P0, P4, and P28) were pooled for three biological replicates, respectively, which were further processed for construction of RNA-seq libraries. Specifically, to satisfy the requirements for sequencing, 15 gonads (i.e., left gonads from 15 individual females) were pooled for each replicate on E15, while only 3 gonads were pooled for each on P0, P4, and P28. Total RNA was extracted using Trizol reagent (Invitrogen, Carlsbad, CA, United States) and treated with DNase I (Invitrogen, Carlsbad, CA, United States) following the manufacturers’ protocol. The libraries were prepared using the Illumina TruSeq mRNA Sample Preparation Kit (Illumina, San Diego, CA, United States) following the manufacture’s recommendation and were sequenced on an Illumina Hiseq X-Ten platform.

The sequencing quality was assessed with both FastQC v0.11.8 and Trimmomatic v0.36 software and the clean reads were obtained by removing the adaptor sequences, reads with > 5% ambiguous bases, and low-quality reads containing > 20% bases with a *Q*-value < 20%. The clean reads were then aligned to the goose reference genome^1^ using the Spliced Transcripts Alignment to a Reference (STAR v2.6.0c) software. The mRNA abundance was expressed as the fragments per kilobase of exon per million fragments mapped (FPKM) using the htseq-count tool from the HTSeq library, and differentially expressed genes (DEGs) between pairwise comparisons were identified using the DESeq2 (v1.16.0) package in R (v3.4.0) software, under the criteria of |log2 fold change (FC)| > 1 and false discovery rate (FDR) < 0.05. Principal component analysis (PCA), hierarchical clustering, and volcano plots were created using R (v3.4.0). The Gene Ontology (GO) and Kyoto Encyclopedia of Genes and Genomes (KEGG) enrichment analyses of DEGs were performed based on the GO^2^ and KEGG^3^ databases, respectively, and significant GO and KEGG terms were identified using the Fisher’s exact test.

### ATAC-Seq Library Preparation, Sequencing, and Analysis

Two biological replicates for each of the E15, P0, P4, and P28 stages, prepared in the same way as RNA-seq, were processed for construction of ATAC-seq libraries using the methods previously described in Buenrostro et al. (2015) and Corces et al. (2017). In brief, cells were dissociated from each pooled sample to obtain single-cell suspensions. Then, cells were suspended in nuclear isolation buffer and washed repeatedly using nuclear wash buffer following a standard nuclear isolation protocol. A total of 50,000 nuclei were pelleted and re-suspended with transposase for 30 min at 37°C. The transposed DNA was purified with a MinElute Kit (Qiagen Inc., Valencia, CA, United States) and was used to generate the library via PCR amplification. All libraries were purified using a Qiagen MinElute PCR Purification Kit following the manufacturer’s instruction and were sequenced on an Illumina Hiseq X-Ten platform.

After removal of the adaptor sequences, the reads were aligned to the goose reference genome^3^ using the Burrows-Wheeler Aligner (BWA 0.7.13-r1126) software. Distribution of the fragment length of unique reads mapping to a single genomic location in a bam file was analyzed for each sample using the bamPEFragmentSize tool. The model-based analysis of ChIP-seq (MACS2 v2.1.2) was applied to call the ATAC-seq peak regions of each sample by using a bam file of uniquely mapped reads as the input, and the *q*-value cutoff for peak calling was 0.05. PCA was performed based on the signals of merged peaks from all samples using R (v3.4.0). Peak annotation was performed by HOMER v4.9.1 function *annotatePeaks.pl*, which picks putative target genes that are located within ATAC-seq called peaks or contain the transcription start sites (TSSs) nearest to these peaks. Motif analysis on peak regions was performed by HOMER v4.9.1 function *findMotifsGenome.pl*. Distribution of uniquely mapped reads resulting from ATAC-seq in a bigwig file across either peaks or gene body was analyzed using deeptools v3.2.1. To identify differential peaks between different stages of development, the ATAC-seq peaks of each sample were merged to generate a consensus set of unique peaks, among which the number of peaks was further counted for each sample using bedtools v2.25.0, and differential peaks were identified by DESeq2 (v1.16.0), with the thresholds of | log2FC| > 1 and *P* < 0.05. GO and KEGG enrichment analyses of the genes around the ATAC-seq peaks were performed as previously described in RNA-seq. For integrative analysis of the ATAC-seq and RNA-seq data, the gain or loss of ATAC-seq peaks within ± 100 kb of TSSs was analyzed in all DEGs between pairwise comparisons. Specifically, the commonly shared peaks by two biological replicates for each developmental stage were firstly obtained by IDR v2.0.3 software with default parameters, and were then used to identify developmental stage-unique peaks by bedtools v2.25.0 with default parameters, followed by peak annotation using HOMER v4.9.1 function *annotatePeaks.pl*. In addition, the DEGs containing differential peaks among three pairwise comparisons were also identified and subjected to functional enrichment analysis. Genomic views of the ATAC-seq data for the selected DEGs were analyzed using the Sushi. R package in R (v3.4.0).

### Quantitative Real-Time PCR (qRT-PCR) Analysis

For qRT-PCR validation of our sequencing data, approximately 1 μg of total RNA was reversed transcribed using the PrimeScript^TM^ RT reagent Kit with gDNA Eraser (Takara Biotechnology Co., Ltd., Dalian, China) according to the manufacturer’s instruction. The PCR reactions were performed on the CFX96^TM^ Real-Time PCR Detection System (Bio-Rad, United States) using the SYBR Premix Ex Taq^TM^ II (Takara Biotechnology Co., Ltd., Dalian, China). Reactions were conducted under the following conditions: pre-denaturation at 95°C for 5 min, followed by 40 cycles of denaturation at 95°C for 15 s and annealing/extension at corresponding temperature of each primer set for 30 s. The no-template controls and negative controls without reverse transcriptase were also included in all qPCR runs. Target specificity for each primer set was validated by melting curve analyses, and the identity of all amplicons was verified by sequencing. All samples were amplified in triplicate and relative expression levels of target genes were normalized to the reference genes *GAPDH* and β*-ACTIN* using the comparative Cq method (ΔΔCq) (Schmittgen and Livak, 2008), and the quantitative data are expressed as the mean ± SEM of three pooled ovaries per group. The qRT-PCR primers of selected genes and transcription factors are listed in Supplementary Table S1.

## Results

### Histomorphological and Immunohistochemical Changes During Early Ovarian Development

**FIGURE 1:**
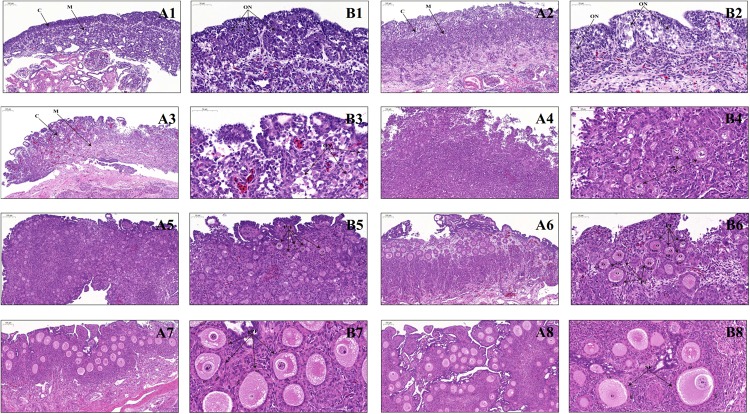
Ovarian histology of geese during late embryonic and early post-hatching development. **(A1–A8)** Low-magnification photomicrograph of HE-stained ovaries from the E15, E26, P0, P4, P7, P14, P21, and P28 geese, respectively. C, ovarian cortex; M, ovarian medulla; Scale bar: 100 μm. **(B1–B8)** Higher-magnification photomicrograph of HE-stained ovaries from the E15, E26, P0, P4, P7, P14, P21, and P28 geese, respectively. ON, oocyte nest; PrF, primordial follicle; PF, primary follicle; gPF, growing primary follicle; SF, secondary follicle; O, primary oocyte; GC, granulosa cells; sTC, single-layered theca cells; dTC, double-layered theca cells; Scale bar: 50 μm.

Morphological observations showed that bilateral ovaries of geese embryos have a similar appearance on E6, show differences on E12, and are clearly different since E15, as manifested by the increasing size and weight of the left ovary in contrast to the gradually shrinking right one throughout development. To further determine histological changes during early stages of development, the left ovaries of embryonic (E6, E12, E15, and E26) and early post-hatching (P0, P4, P7, P14, P21, and P28) geese were stained with HE and microscopically inspected. Our results showed that the E6 ovary looking like a strip shape overlies on the ventral surface of mesonephros and contains several germ cells that are characterized by their large size, and since E12, the ovary consists of an outer dense cortex and an inner sparse medulla (data not shown). From E15 to E26, the ovarian cortex becomes more thickened, and moreover, germ cells differentiate into primary oocytes with an enlarged volume and form more oocyte nests (Figure 1). In particular, since PAS can efficiently stain glycogens that are abundant in the cytoplasm of germ cells and CVH is recognized as the most reliable germ cell marker (Kim and Han, 2018), both PAS staining and immunohistochemical staining with CVH were used to reveal changes in the number of germ cells during late geese embryonic development. It was observed that the total number of primary oocytes within the nests decreases from E15 to E26 (Supplementary Figure S1). Furthermore, as shown in Figure 1, immediately after hatching (P0), both the thickness of the ovarian cortex and the oocyte volume increase, but the oocytes are still present in the form of nests. On P4, several oocyte nests start to break down, and the separated oocytes are surrounded by a single layer of somatic pre-granulosa cells, forming the primordial follicles. Thereafter, the structure of primordial follicles becomes more pronounced and their number remarkably increases on P7, accompanied by increased follicular size and multiplied granulosa cells. With the enlarged oocyte diameter as well as the transition of granulosa cells from a flattened to a columnar shape, primordial follicles develop into primary follicles on P14. A single layer of theca cells arranges outside of granulosa cells on P21, and moreover, the single theca layer differentiates into a double-layered structure (including theca externa and interna) and the granulosa layer becomes multilayered on P28, leading to the formation of secondary follicles. Taken together, these results suggested that in the goose, ovary peri-hatching oocyte loss, primordial follicle formation, and the primordial-to-growing (primary and secondary) follicle transition take place during the periods from E15 to P0, from P0 to P4, and from P4 to P28, respectively.

### Genome-Wide Gene Expression Dynamics During Early Ovarian Development

To determine global gene expression profiles during early ovarian development, three mRNA pools from each of four representative developmental stages (i.e., E15, P0, P4, and P28) were subjected to RNA-seq. As shown in Supplementary Table S2, more than 8.2 billion clean bases and 27.4 million clean reads were yielded by each library; the Q20 ratio, Q30 ratio, and GC content varied from 97.46 to 98, 93.27 to 94.62, and 50.26 to 51.23%, respectively, and 75.2 to 79.3% clean reads from each library were uniquely mapped to the reference goose genome. PCA analysis showed that 12 RNA-seq libraries were sorted into three distinct clusters corresponding to E15, P0 and P4, and P28, and among them, P0 and P4 shared more similar expression patterns partially due to shorter developmental interval (Supplementary Figure S2A). In spite of this, relatively higher similarity in global gene expression profiles among three biological replicates for each developmental stage indicated good reproducibility of our sequencing data.

**FIGURE 2:**
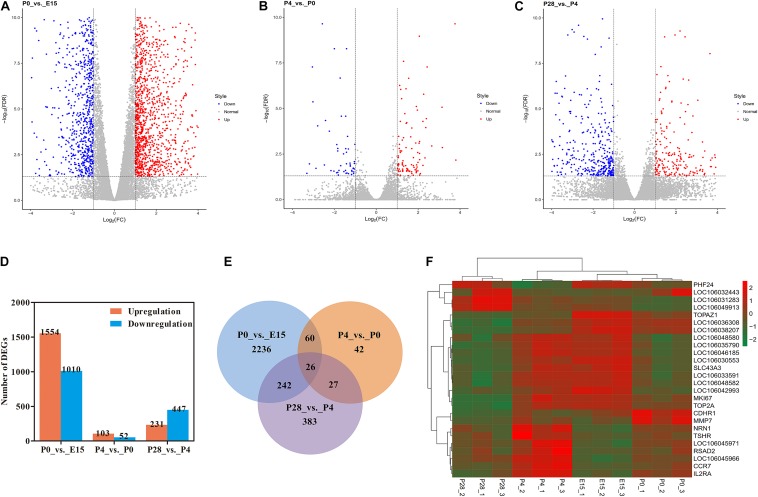
RNA-seq reveals transcriptome dynamics during early ovarian development. **(A–C)** Volcano plots showing significantly up- and downregulated genes between P0 vs. E15, P4 vs. P0, and P28 vs. P4, respectively. **(D)** The number of differentially expressed genes (DEGs) for each pairwise comparison. **(E)** Venn diagram indicating the number of DEGs between three pairwise comparisons. **(F)** Heatmap of the 26 DEGs overlapped among three pairwise comparisons.

FPKM-based quantitative analysis was employed to reveal dynamic expression changes of all identified genes between different stages of early ovarian development (Figures 2A–C). Of them, significantly up- and downregulated genes were screened for each pairwise comparison according to the same thresholds (Figure 2D). In detail, 1554 up- and 1010 downregulated genes were identified between P0 vs. E15, 103 and 52 genes were up- and downregulated between P4 vs. P0, and 231 up- and 447 downregulated genes were identified between P28 vs. P4, respectively. As shown in Figure 2E, there were 26 differentially expressed genes (DEGs) present between all pairwise comparisons. Meanwhile, 86 genes were found to be differentially expressed during the period from E15 to P4, and 53 DEGs were commonly identified between the comparisons of P4 vs. P0 and P28 vs. P4. Hierarchical clustering analysis suggested that developmental stage had significant effects on the expression levels of these 26 DEGs and confirmed that more similar expression patterns existed among three biological replicates for each stage. In addition, E15 and P0 were first clustered, and then were successively clustered with P4 and P28. Three major clusters of genes, which shared more similar expression patterns throughout early ovarian development, were also observed (Figure 2F). These data revealed dynamic changes in the ovarian transcriptome during late embryonic and early post-hatching stages, with the most extensive changes during peri-hatching oocyte loss.

### Enrichment Analysis of Differentially Expressed Genes During Early Ovarian Development

**FIGURE 3:**
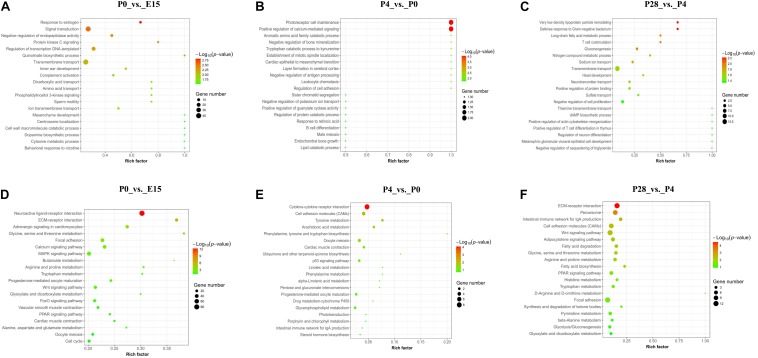
GO and KEGG analyses of DEGs between different stages of early ovarian development. **(A–C)** Top 20 GO categories of biological process enriched by DEGs between P0 vs. E15, P4 vs. P0, and P28 vs. P4, respectively. **(D–F)** Top 20 KEGG pathways enriched by DEGs between P0 vs. E15, P4 vs. P0, and P28 vs. P4, respectively.

The top 20 GO categories of biological process (BP) enriched by DEGs between different stages of early ovarian development were displayed in Figures 3A–C. For DEGs identified between P0 vs. E15, most of them were enriched in the terms associated with transmembrane transport, signal transduction, regulation of transcription (DNA-templated), and negative regulation of endopeptidase activity, and several significantly enriched terms were related to regulation of ovarian cell functions (response to estrogen, protein kinase C signaling, phosphatidylinositol 3-kinase signaling, and dopamine biosynthetic process). Likewise, the top 2 mostly enriched terms by DEGs between P4 vs. P0 were photoreceptor cell maintenance and positive regulation of calcium-mediated signaling, while those between P28 vs. P4 were transmembrane transport and negative regulation of cell proliferation. Besides, several terms associated with ovarian tissue remodeling (establishment of mitotic spindle localization, layer formation in cerebral cortex, negative regulation of antigen processing, regulation of cell adhesion, and response to retinoic acid) were significantly enriched between P4 vs. P0, while those related to lipid metabolism (very-low-density lipoprotein particle remodeling, long-chain fatty acid metabolic process, gluconeogenesis, and negative regulation of sequestering of triglyceride) were identified between P28 vs. P4. Of note, one term, namely, photoreceptor cell maintenance, was commonly enriched by DEGs from P0 vs. E15 and P4 vs. P0, and the two commonly involved DEGs were *CDHR1* and *RP1L1*. Between the comparisons of P4 vs. P0 and P28 vs. P4, three commonly enriched terms were identified, including tryptophan catabolic process to kynurenine, layer formation in cerebral cortex, and defense response to gram-negative bacterium, and only one commonly involved DEG (i.e., *IDO2*, *LRP8*, and *MMP7*) was identified for each term, respectively (Supplementary Table S3). It was thus summarized that most of the DEGs between these pairwise comparisons were enriched in different GO-BP terms, with transmembrane transport and signal transduction, photoreceptor cell maintenance and tissue remodeling, and transmembrane transport and lipid metabolism being the most enriched terms for P0 vs. E15, P4 vs. P0, and P28 vs. P4, respectively.

Figures 3D–F presented the top 20 KEGG pathways enriched by DEGs between different stages of early ovarian development. The top 3 mostly enriched pathways by DEGs between P0 vs. E15 were neuroactive ligand–receptor interaction, MAPK signaling, and focal adhesion, those between P4 vs. P0 were cytokine–cytokine receptor interaction, cell adhesion molecules (CAMs), and oocyte meiosis, and those between P28 vs. P4 were ECM–receptor interaction, Wnt signaling, and focal adhesion. Besides, other pathways associated with oocyte maturation (progesterone-mediated oocyte maturation, oocyte meiosis, FoxO signaling, and Wnt signaling) were identified between P0 vs. E15, and those related to lipid metabolism (peroxisome, adipocytokine signaling, fatty acid degradation, fatty acid biosynthesis, PPAR signaling, glycolysis, and gluconeogenesis) were identified between P28 vs. P4. Of note, one KEGG pathway, namely, oocyte meiosis, was commonly enriched by DEGs from P0 vs. E15 and P4 vs. P0, and the only one commonly involved DEG was *SGOL1*. Between the comparisons of P4 vs. P0 and P28 vs. P4, only one commonly enriched pathway was identified, namely, CAMs, and the two commonly involved DEGs were *PTPRC* and *CD2* (Supplementary Table S3). It was thus summarized that most of DEGs between these pairwise comparisons were enriched in different KEGG pathways, with neuroactive ligand–receptor interaction and oocyte maturation, tissue remodeling and metabolic processes, and signal transduction and lipid metabolism being the most enriched pathways for P0 vs. E15, P4 vs. P0, and P28 vs. P4, respectively.

### Establishing the Open Chromatin Landscape During Early Ovarian Development

**FIGURE 4:**
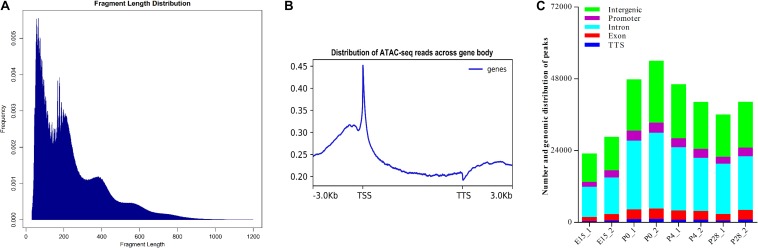
Quality estimation, peak calling, and genomic distribution of ATAC-seq reads during early ovarian development. **(A)** Frequency distribution of fragment lengths within a representative ATAC-seq library. The smallest fragment peaks represent sequencing reads in inter-nucleosome open chromatin, while larger peaks represent those spanning nucleosomes. **(B)** Distribution plot of sequencing reads from a representative ATAC-seq library across all genes. We normalized all genes according to their lengths and calculated the average ATAC-seq signals between TSS (–3 kb) and TTS (+ 3 kb) of all genes after peak calling. **(C)** Number and genomic distribution of peaks identified by ATAC-seq in each sample. Genomic annotations included the promoter (± 1 kb of TSS), TTS (between –100 and + 1 kb of TTS), exon, intron, and intergenic region, and the front region was regarded as the final annotation according to the above order if overlap occurred.

To determine the chromatin accessibility landscape during early ovarian development, two pooled ovarian cell suspensions from each of four representative developmental stages (i.e., E15, P0, P4, and P28) were subjected to ATAC-seq. As shown in Supplementary Table S4, more than 11.0 billion clean bases and 46.6 million clean reads were yielded by each library; the Q20 ratio, Q30 ratio, and GC content varied from 91.62 to 93.69, 84.59 to 87.66, and 45.03 to 47.51%, respectively, and 83.8 to 87.6% clean reads from each library were uniquely mapped to the reference goose genome. PCA analysis showed that there were generally higher similarities in the chromatin accessibility landscape between two biological replicates of each developmental stage, with the maximal similarity between those of P28, and that the ATAC-seq profiles of P0 showed a relatively large difference in comparison with those of all other stages (Supplementary Figure S2B). Furthermore, as expected, the majority of fragments within each library had a shorter length and represented the inter-nucleosome open chromatin, while fragments of > 147 bp indicated sequencing reads spanning ≥ 1 nucleosomes (Figure 4A). Distribution of the average ATAC-seq signals across all genes showed that there were strong signals present around TSSs (Figure 4B), suggesting that the majority of ATAC-seq reads was distributed around TSSs. Hence, these results demonstrated the reliability and high quality of our ATAC-seq data. As shown in Figure 4C, MACS2-based quantitative analysis showed that there were 23,020 and 28,570 peaks present in two biological replicates for E15, 47,792 and 54,045 peaks in those for P0, 46,186 and 40,236 peaks in those for P4, and 36,083 and 40,273 peaks in those for P28, which was indicative of good reproducibility in the biological samples for each developmental stage. Furthermore, peak annotation suggested that 42.74–48.09% of peaks from all samples were located in introns, 35.84–41.25% in intergenic regions, 6.34–8.54% in promoters (±1 kb of TSS), 5.77–8.01% in exons, and 1.85–2.34% in the range between –100 bp and + 1 kb of transcription termination site (TTS). Notably, similar genomic distribution patterns of peaks were observed between two biological replicates for each developmental stage, which again demonstrated the reliability and good reproducibility of our ATAC-seq data.

### Dynamic Changes in Open Chromatin During Early Ovarian Development

**FIGURE 5:**
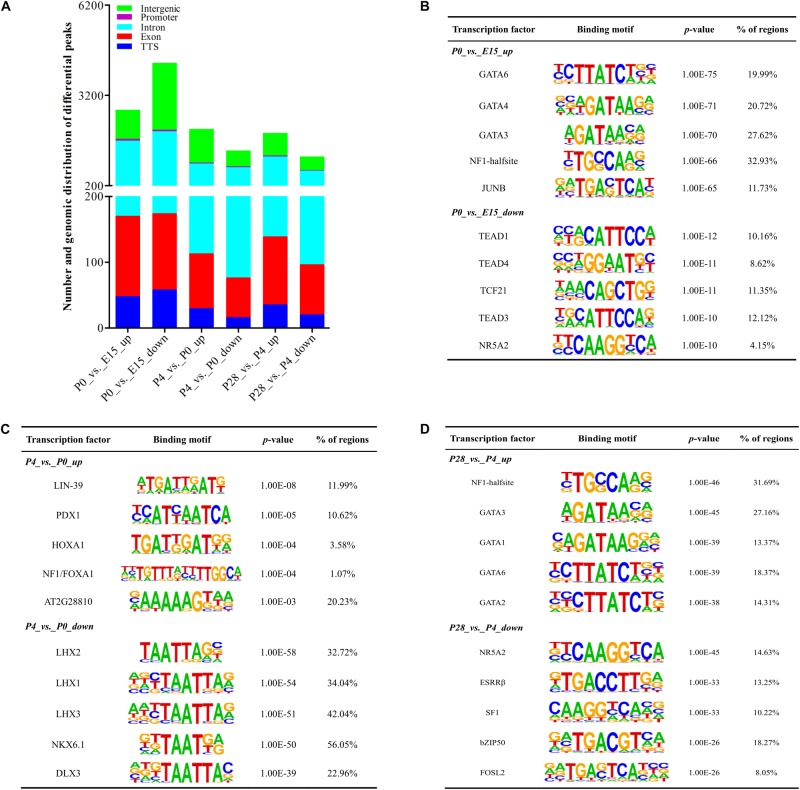
Dynamic changes of accessible chromatin and motif analysis of differential peaks during early ovarian development. **(A)** Number and genomic distribution of significantly increased and decreased peaks between different stages of early ovarian development. **(B–D)** Top 5 transcription factor binding motifs enriched in significantly increased and decreased peak regions according to the *p*-values between P0 vs. E15, P4 vs. P0, and P28 vs. P4, respectively.

To reveal how the accessibility of chromatin changes during early ovarian development, we compared differences in the identified peaks between different developmental stages using DEseq2. As shown in Figure 5A, compared to ovaries of E15, 2723 increased and 4279 decreased peaks were detected in those of P0. Likewise, 2083 and 1367 peaks were increased and decreased between P4 vs. P0, while 1951 increased and 1167 decreased peaks were identified between P28 vs. P4. These results demonstrated that the most extensive changes in accessible chromatin regions of the ovary take place during peri-hatching oocyte loss, which is similar to its transcriptome dynamics, supporting the notion that differential chromatin accessibility may lead to developmental stage-dependent gene expression profiles. Furthermore, 56.12, 35.45, 2.16, 4.5, and 1.77% of increased peaks between P0 vs. E15 were annotated in introns, intergenic regions, promoters, exons, and TTS, respectively, while 42.88, 51.89, 1.15, 2.71, and 1.37% of decreased peaks between P0 vs. E15 were successively present in the above regions. As to differential peaks between P4 vs. P0, 39.5, 53.61, and 1.44% of increased peaks while 53.9, 38.13, and 2.33% of decreased peaks were located in introns, intergenic regions, and promoters, respectively. Meanwhile, 52.57, 38.45, and 1.84% of increased peaks while 51.63, 38.13, and 1.94% of decreased peaks between P28 vs. P4 were present in introns, intergenic regions, and promoters, respectively. Therefore, it was inferred that differences in the genomic distribution of both increased and decreased peaks during early ovarian development may facilitate chromatin-level regulation of gene expression.

Figures 5B–D presented the top 5 significantly enriched transcription factor binding motifs within differential peak regions between different developmental stages. For differential peaks between P0 vs. E15, the top 5 motifs enriched by increased peaks were known as binding sites of GATA6, GATA4, GATA3, NF1-halfsite, and JUNB, while those enriched by decreased peaks contained binding sites for TEAD1, TEAD4, TCF21, TEAD3, and NR5A2. Similarly, the top 5 binding sites enriched by increased and decreased peaks between P4 vs. P0 were those for LIN-39, PDX1, HOXA1, NF1/FOXA1, and AT2G28810, and those for LHX2, LHX1, LHX3, NKX6.1, and DLX3, respectively. Increased and decreased peaks between P28 vs. P4 were significantly enriched for binding sites of NF1-halfsite, GATA3, GATA1, GATA6, and GATA2, and for those of NR5A2, ESRRβ, SF1, bZIP50, and FOSL2, respectively. Differences in the availability of these potential binding sites for respective transcriptional factors were mainly attributed to differential chromatin accessibility, and these identified transcription factors could play important but differential roles in regulation of peri-hatching oocyte loss, primordial follicle formation, and the primordial-to-secondary follicle transition.

### Enrichment Analysis of Nearby Genes of Differential Open Chromatin Regions During Early Ovarian Development

The top 20 KEGG pathways enriched by genes containing the nearest TSSs to differential peaks between different stages of early ovarian development are shown in Supplementary Figure S3. As for the comparison between P0 vs. E15, the top 3 mostly enriched pathways by nearby genes of increased peaks were focal adhesion, mTOR signaling, and FoxO signaling, while those by nearby genes of decreased peaks were neuroactive ligand–receptor interaction, calcium signaling, and MAPK signaling (Supplementary Figures S3A,B). Likewise, most of the genes containing the nearest TSSs to increased peaks between P4 vs. P0 were enriched in neuroactive ligand–receptor interaction, Wnt signaling, and gap junction, while those located nearby decreased peaks between P4 vs. P0 were enriched in focal adhesion, ErbB signaling, and ECM–receptor interaction (Supplementary Figures S3C,D). With regard to the comparison between P28 vs. P4, the top 3 mostly enriched pathways by nearby genes of increased and decreased peaks were Wnt signaling, calcium signaling, and TGFβ signaling, and regulation of actin cytoskeleton, Wnt signaling, and NOD-like receptor signaling, respectively (Supplementary Figures S3E,F). Besides, other pathways associated with oocyte maturation (phosphatidylinositol signaling, adherens junction, apoptosis, CAMs, and Wnt signaling) were identified between P0 vs. E15, and those related to metabolic processes (mucin type O-glycan biosynthesis, tryptophan metabolism, butanoate metabolism, pentose and glucuronate interconversions, SNARE interactions in vesicular transport, and TGFβ signaling) were identified between P4 vs. P0, and those associated with lipid metabolism (phosphatidylinositol signaling system, phosphonate and phosphinate metabolism, pantothenate and CoA biosynthesis, and fatty acid elongation) were identified between P28 vs. P4. It was thus summarized that most nearby genes of differential peaks between these pairwise comparisons were enriched in different KEGG pathways, with signal transduction and oocyte maturation, ligand–receptor interaction and metabolic processes, and signal transduction and lipid metabolism being the most enriched pathways for P0 vs. E15, P4 vs. P0, and P28 vs. P4, respectively.

### Correlation of Chromatin Accessibility With Dynamic Transcriptome Changes During Early Ovarian Development

**FIGURE 6:**
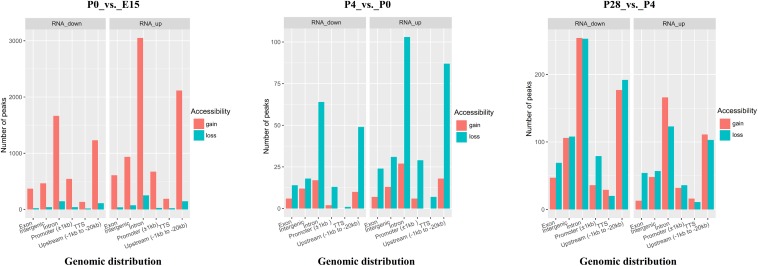
Bar plots indicating the number and genomic distribution of developmental stage-unique peaks around TSSs (±100 kb) of significantly up- and downregulated genes between P0 vs. E15, P4 vs. P0, and P28 vs. P4, respectively. Genomic annotations included the promoter (± 1 kb of TSS), upstream (between –1 and –20 kb of TSS), TTS (between –100 and + 1 kb of TTS), exon, intron, and intergenic region, and the front region was regarded as the final annotation according to the above order if overlap occurred. Gain or loss of accessibility indicates the newly appeared or disappeared open chromatin regions between each pairwise comparison.

To determine whether developmental stage-dependent open chromatin regions are associated with dynamic gene expression patterns, we performed an integrative analysis of the obtained ATAC-seq and RNA-seq data. Analysis of the gain or loss of ATAC-seq peaks within ± 100 kb of TSSs in DEGs between different developmental stages suggested remarkable changes in the number and genomic distribution of developmental stage-unique peaks during early ovarian development (Figure 6). Specifically, the largest number of either the newly appeared or disappeared open chromatin regions was identified around TSSs of significantly up- or downregulated genes between P0 vs. E15, followed by the comparison of P28 vs. P4 and P4 vs. P0, respectively. As for either up- or downregulated genes between P0 vs. E15, the number of peaks unique to P0 was far more than that of peaks unique to E15. In contrast, compared to P0, less open chromatin regions around TSSs of up- or downregulated genes were detected in P4, while almost equal numbers of the newly appeared and disappeared open chromatin regions were found in both up- and downregulated genes between P28 vs. P4. Furthermore, although most of the developmental stage-unique peaks within ± 100 kb of TSSs in DEGs among three pairwise comparisons were distributed in intronic and upstream regions, the genomic distribution proportion of all these peaks differed according to developmental stage and the pattern of gene regulation. Regarding the promoter region, most of accessible chromatin was gained from E15 to P0 but was lost from P0 to P4, whereas the newly appeared and disappeared peaks were almost equally distributed around TSSs of upregulated genes but were differently distributed around those of downregulated genes between P28 vs. P4. Compared to P0, there were not P4-unique peaks distributed around TTSs of up- or downregulated genes. Of note, as for the intronic and upstream regions, a larger number of peaks were present in up- than downregulated genes between E15 vs. P0 and P4 vs. P0, but the opposite was seen between P28 vs. P4. Taken together, these results could further reinforce the notion that developmental stage-dependent gene expression profiles are regulated at the chromatin level by modulating the accessibility of different functional genomic regions to transcription factors.

**FIGURE 7:**
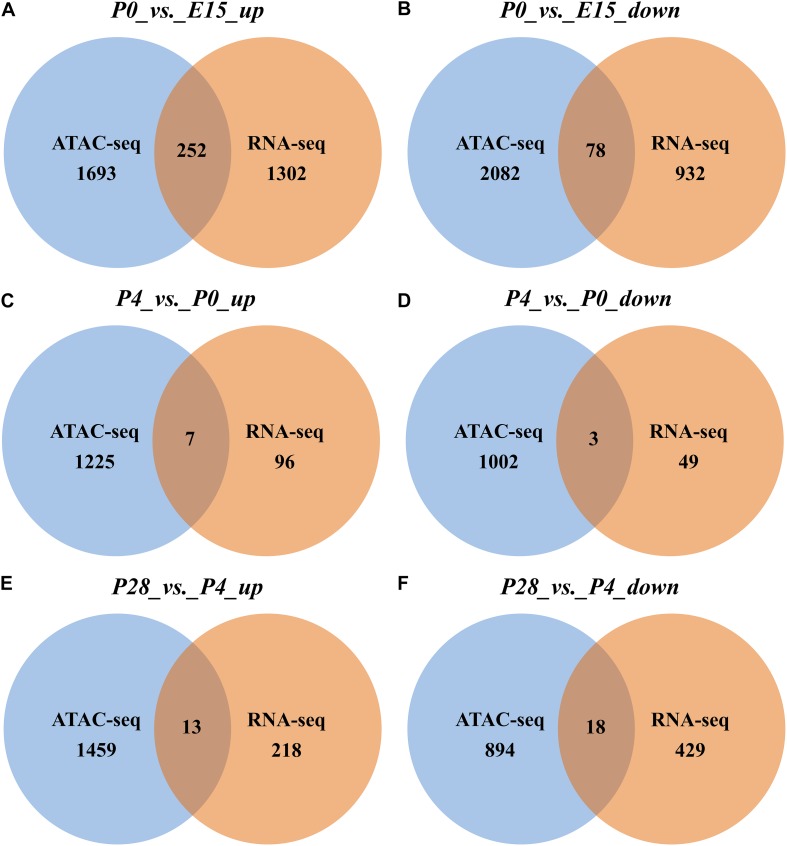
Venn diagrams showing the number of nearby DEGs of differential peaks between different stages of early ovarian development. **(A,C,E)** Overlap of significantly upregulated genes identified by RNA-seq with nearby genes of significantly increased ATAC-seq peaks between P0 vs. E15, P4 vs. P0, and P28 vs. P4, respectively. **(B,D,F)** Overlap of significantly downregulated genes identified by RNA-seq with nearby genes of significantly decreased ATAC-seq peaks between P0 vs. E15, P4 vs. P0, and P28 vs. P4, respectively.

In addition, we also identified the DEGs containing associated developmental stage-selective peaks among three pairwise comparisons. As shown in Figures 7A,B, in comparison with ovaries of E15, 252 genes that were upregulated in those of P0 had the nearest TSSs to increased peaks, accounting for ∼16.2% of all upregulated genes, while 78 downregulated genes (∼7.7%) located nearest to decreased peaks. In contrast, seven upregulated (∼6.8%) and three downregulated (∼5.8%) genes contained the nearest TSSs to increased and decreased peaks between P4 vs. P0, respectively (Figures 7C,D), while 13 upregulated (∼5.6%) and 18 downregulated (∼4%) genes located nearest to increased and decreased peaks between P28 vs. P4, respectively (Figures 7E,F). Among them, developmental stage-associated changes in the ATAC-seq profiles of several selected genes, including two DEGs (i.e., *INSR* and *TTK*) between P0 vs. E15, one (i.e., *KL*) between P4 vs. P0, and one (i.e., *GK*) between P28 vs. P4, were also displayed (Supplementary Figure S4). These results suggested that altered gene expression levels could be correlated with dynamic changes of chromatin accessibility during early ovarian development.

KEGG pathways enriched by the DEGs containing the nearest TSSs to differential peaks between different developmental stages were shown in Supplementary Figure S5. As for the comparison between P0 vs. E15, the top 3 mostly enriched pathways by upregulated genes around increased peaks were neuroactive ligand–receptor interaction, MAPK signaling, and FoxO signaling, while those by downregulated genes around decreased peaks were oocyte meiosis, cell cycle, and base excision repair (Supplementary Figures S5A,B). Likewise, between P4 vs. P0, upregulated genes around increased peaks were enriched in glycerolipid and glycerophospholipid metabolism, while downregulated genes around decreased peaks were enriched in pentose and glucuronate interconversions (Supplementary Figures S5C,D). With regard to the comparison between P28 vs. P4, the significantly enriched pathways by upregulated genes around increased peaks only included Wnt signaling, while those by downregulated genes around decreased peaks included pantothenate and CoA biosynthesis, β-alanine metabolism, drug metabolism (other enzymes), glycerolipid metabolism, and pyrimidine metabolism (Supplementary Figures S5E,F). It was thus summarized that different KEGG pathways were enriched by nearby DEGs of differential peaks between different developmental stages, with neuroactive ligand–receptor interaction and MAPK signaling, glycerolipid metabolism, and Wnt signaling and metabolic processes being the mainly enriched pathways for P0 vs. E15, P4 vs. P0, and P28 vs. P4, respectively.

### Validation of Expression Profiles of Key Candidate Genes Involved in Early Ovarian Development

**FIGURE 8:**
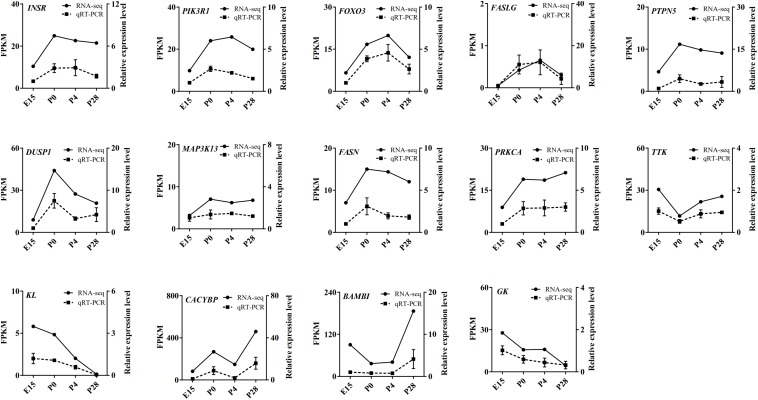
qRT-PCR validation of expression of the main DEGs involving the MAPK signaling, FoxO signaling, cell cycle, lipid metabolism, and Wnt signaling pathways in the geese ovaries during late embryonic and early post-hatching stages. The qRT-PCR results are expressed as the mean ± SEM of 3 pooled ovaries per group and signified by the dashed line, while the RNA-seq data are signified by the solid line.

**FIGURE 9:**
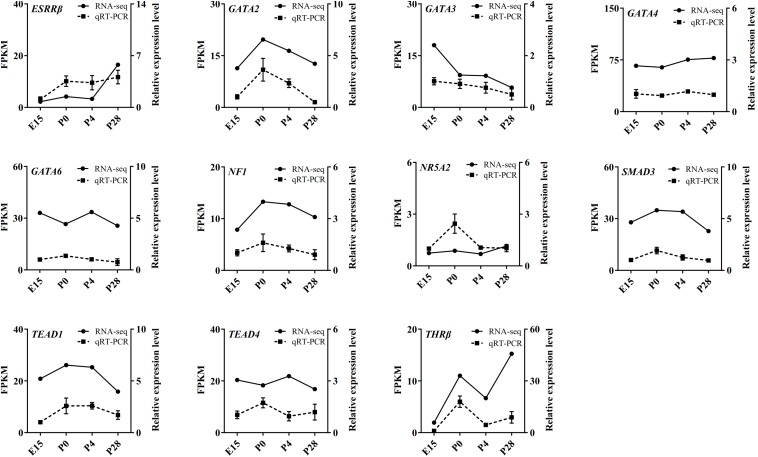
qRT-PCR validation of expression of several transcription factors identified by ATAC-seq in the geese ovaries during late embryonic and early post-hatching stages. The qRT-PCR results are expressed as the mean ± SEM of three pooled ovaries per group and signified by the dashed line, while the RNA-seq data are signified by the solid line.

Based on our RNA-seq and ATAC-seq analyses, subsets of the DEGs involved in the MAPK signaling, FoxO signaling, cell cycle, lipid metabolism, and Wnt signaling pathways (including *INSR*, *FASLG*, *PI3KR1*, *FoxO3*, *PTPN5*, *DUSP1*, *MAP3K13*, *FASN*, *PRKCA*, *TTK*, *KL*, *CACYBP*, *BAMBI*, and *GK*) and the identified transcription factors (including *ESRR*β, *GATA2*, *GATA3*, *GATA4*, *GATA6*, *NF1*, *NR5A2*, *SMAD3*, *TEAD1*, *TEAD4*, and *THR*β) were selected for qRT-PCR validation. As shown in Figures 8, 9, regardless of differences in the magnitude of fold-changes, expression of almost all these selected mRNAs determined by qRT-PCR displayed changes in the same direction with that observed using RNA-seq, indicating the true reliability of our Illumina sequencing methods.

## Discussion

In contrast to the situation in mammals, mechanisms responsible for early avian ovarian development remain poorly understood. In this study, histological, transcriptomic, and chromatin accessibility dynamics were determined in geese ovaries during the periods from E6 to P28. As in chicken embryos (Matova and Cooley, 2001; Guioli et al., 2014), the gonads of female geese originally present an epithelial-like morphology and are primarily composed of germ cells. As development proceeds, the left ovary increases in its thickness and is organized into the cortex and medulla portions since E12. Oocyte nest forms as early as E15, remains throughout embryogenesis, and undergoes breakdown to assembly primordial follicles on P4. Of note, the total number of primary oocytes decreases from E15 to E26 in companion with their enlarged volume. Besides, the primordial to primary follicle transition occurs on P14, while primary follicles develop into secondary follicles on P28. Thus, our results demonstrated that a series of key molecular events, including peri-hatching oocyte loss, oocyte nest breakdown and primordial follicle formation, and the primordial-to-growing follicle transition, take place in the goose ovary during late embryonic and early post-hatching stages.

Although understanding how gene regulatory networks control the orderly progression of these events remains a long-standing challenge, recent advances in determining dynamic gene expression and chromatin accessibility changes in human and mouse ovarian tissues and cells using next generation sequencing-based epigenomic techniques (e.g., RNA-seq, CHIP-seq, DNase-seq, and ATAC-seq) are contributing to the identification of key genes, *cis*-regulatory elements, and organism-specific regulatory systems (Apostolou and Hochedlinger, 2013; Barragan et al., 2017; Guo et al., 2017; Yu et al., 2017; Miyamoto et al., 2018; Gu et al., 2019). Therefore, we integrated RNA-seq and ATAC-seq to unravel the transcriptional networks regulating early avian ovarian development. Our results showed that the most extensive changes in both the ovarian transcriptome and chromatin accessibility took place during the transition from mid- to late embryogenesis (P0 vs. E15), indicating that transcriptional activity of target genes is strongly associated with the accessibility of functional genomic regions (i.e., transcription factor occupancy) that is finely tuned at the chromatin level, which was concordant with previous findings in a range of vertebrate species (Stergachis et al., 2014; Ackermann et al., 2016; Lowdon et al., 2016; Miyamoto et al., 2018; Gu et al., 2019). As revealed by ATAC-seq analysis, the majority (>90%) of developmental stage-selective peaks was present in intronic and intergenic regions, whereas less than 4% of peaks occurred in regions around TSS and TTS. Our data together with the results from previous studies (Stergachis et al., 2014; Guo et al., 2017; Miyamoto et al., 2018) strengthened the notion that transcriptional changes during oogenesis and folliculogenesis are mainly regulated by non-protein-coding regions, particularly the distal regulatory elements from the gene locus itself. Furthermore, comparison of the genomic distribution of increased and decreased peaks between either P0 vs. E15 or P4 vs. P0 revealed proportional differences in intronic and intergenic regions as well as in promoters; however, much less variation was seen between P28 vs. P4. As only 26 DEGs were commonly identified among three pairwise comparisons, it was postulated that chromatin-level regulation of gene expression differ at different stages of early ovarian development and that developmental stage-selective gene regulation depend on the accessibility of their different regulatory elements. In support of this, analysis of appearance of the ATAC-seq peaks around DEGs among three pairwise comparisons suggested that the greatest changes in the number of developmental stage-unique peaks occurred between P0 vs. E15, which was consistent with our above results. In addition, the number of the newly appeared and disappeared peaks around DEGs significantly changed between three pairwise comparisons, and moreover, the genomic distribution proportions of these peaks were also variable depending on the stage of ovarian development and the style of gene regulation. Nevertheless, it should be noted that only a small fraction of DEGs showed differential chromatin accessibility between different stages of early ovarian development. Here, we considered several possible reasons for this observation: (1) genes change their expression possibly by binding of different transcription factor combinations to equally accessible sites, (2) differentially accessible sites do not result in changes in bulk RNA levels but in cell type-specific patterns, and/or (3) there may exist still other undefined mechanisms except for chromatin reorganization regulating gene expression during early ovarian development.

It is well known that transcription factors control developmental stage-dependent gene expression programs through binding with their featured motifs in the genome and that highly compacted chromatin architecture often impedes the access of transcription factors to distinct functional genomic regions (Kagey et al., 2010). Considering that ATAC-seq detects Tn5 transposase accessible chromatin regions (potential transcription factor binding sites) with higher sensitivity (Buenrostro et al., 2013), motif analysis of increased and decreased peaks provided new insights into the establishment of developmental stage-selective transcriptional machinery in the avian ovary. As with peak annotation, the identified transcription factor-binding motifs differed remarkably in their sequences and numbers not only between different developmental stages but also between increased and decreased peaks in the same pairwise comparison. Among the predicted transcription factors that contain binding sites in the five most reliable motifs in either increased or decreased peaks between different developmental stages, several members of the GATA family of transcription factors (e.g., GATA2, 3, 4, and 6) have not only been demonstrated to play critical roles in mammalian fetal and postnatal ovarian development (Viger et al., 2008), but they were also reported to be differentially expressed in either the ovaries of embryos and post-hatching chicks or the different sized follicles of laying geese ovaries (Oreal et al., 2002; Carre et al., 2011; Yuan et al., 2014). Likewise, altering levels of SF1, NR5A2, ESRRβ, and several members of the SMAD and LHX families were also detected in the chick ovaries during embryonic to post-hatching transition (Oreal et al., 2002; Ghafari et al., 2007; Carre et al., 2011). In contrast, information about the functions of NF1, THRβ, and the TEAD family remains scarce in the avian ovary, all of which were evidenced to be involved in regulating cell survival, proliferation, and adhesion partially by acting as components of multiple signal transduction (e.g., the Hippo and Ras-MEK/Akt) pathways and some of which were expressed in a variety of tissues of the chick embryos (Schafer et al., 1993; Cao et al., 2008; Haba et al., 2011). Significantly, expression of these transcription factors in embryonic and post-hatching geese ovaries was determined by RNA-seq and qRT-PCR. The consistent results between both methods verified the existence of their mRNAs in the goose ovary and indicated that their differential abundance and respective fluctuating levels throughout development may be implicative of different actions in regulating peri-hatching oocyte loss and primordial follicle formation and development in birds, albeit additional studies needed to elucidate the underlying mechanisms. Of note, members of either the GATA or TEAD families displayed apparent differences in their expression patterns during early goose ovarian development, suggesting their coordinate regulation of developmental stage-selective transcriptional machinery. The physiological relevance of other ATAC-seq identified transcription factors in the avian ovary requires further investigations in future.

At the transcriptomic level, most of the DEGs between P0 vs. E15 were enriched in the GO-BP terms including transmembrane transport, signal transduction, and regulation of transcription, as well as in the KEGG pathways related to neuroactive ligand–receptor interaction, signal transduction, focal adhesion, and oocyte maturation. In the meantime, enrichment analysis showed that a large number of nearby genes of differential peaks between P0 vs. E15 were also enriched in focal adhesion, signal transduction, and neuroactive ligand–receptor interaction. These results altogether suggested that the landscape of accessible chromatin may direct the dynamic changes in expression of genes involved in particular pathways during embryonic development of the ovary. Further evidence to reinforce this notion comes from our integrative analysis of ATAC-seq and RNA-seq results between P0 vs. E15. It revealed that the DEGs exhibiting differential ATAC-seq peaks were significantly enriched in neuroactive ligand–receptor interaction, signal transduction, oocyte meiosis, and cell cycle. Expression of the main DEGs enriched in the MAPK and FoxO signaling pathways as well as one associated with cell cycle were validated by qRT-PCR, because all of these pathways were shown to have essential roles in regulation of mammalian and avian ovarian cell functions (Liu et al., 2007; Johnson and Woods, 2009; Zhao et al., 2018). In accordance with the RNA-seq data, results from qRT-PCR confirmed that both four DEGs (i.e., *INSR*, *FASLG*, *PI3KR1*, and *FoxO3*) in the FoxO- and seven DEGs (i.e., *INSR*, *FASLG*, *PTPN5*, *DUSP1*, *MAP3K13*, *FASN*, and *PRKCA*) in the MAPK pathways showed enhanced mRNA levels during the embryonic to immediate post-hatching transition, whereas the opposite was seen in levels of one cell cycle-related gene (i.e., *TTK*). Hence, it was conceivable that these DEGs could be responsible for oocyte loss during late embryonic development in birds. Also, dramatic changes in their expression patterns from P0 to P28 may suggest potential roles in regulating postembryonic development of the avian ovary.

By comparison, a relatively small number of genes were differentially expressed during oocyte nest breakdown and primordial follicle formation (P4 vs. P0). Several top enriched GO-BP terms and/or KEGG pathways by either these DEGs or genes around differential peaks were related to ligand–receptor interaction, tissue remodeling, signal transduction, and metabolic processes, most of which were reported to be associated with the formation and development of mammalian ovarian primordial follicles (Smith et al., 2002; Nilsson et al., 2010; Hu et al., 2019). Among nearby DEGs of differential chromatin accessibility, only the *SLC2A11* and klotho (*KL*) genes were shown to be possibly involved in regulating mammalian ovarian activities (Mao et al., 2018; Hu et al., 2019) while others have been largely implicated in regulating cancer cell proliferation and apoptosis. Furthermore, our quantitative results verified that the mRNA levels of *KL* decreased continuously during early goose ovarian development, which may be indicative of its inhibitory actions in primordial follicle formation and subsequent progression to secondary follicles. Nevertheless, the exact roles of these DEGs during early ovarian development require further investigations. Regarding the primordial-to-growing follicle transition, most of either the DEGs or genes around differential peaks were enriched in pathways related to tissue remodeling, lipid metabolism, and signal transduction. Of them, glycolipid metabolism-related pathways included peroxisome, adipocytokine signaling, fatty acid degradation, fatty acid biosynthesis, fatty acid elongation, glycolysis, gluconeogenesis, phosphonate and phosphinate metabolism, and pantothenate and CoA biosynthesis, most of which are essential for sustaining mammalian normal ovarian development by regulating oocyte maturation and somatic cell proliferation (Su et al., 2008; Collado-Fernandez et al., 2012). These results implied that cellular glycolipid metabolic status may function as a trigger for primordial follicle development in birds. Furthermore, expression of 3 of the 31 RNA-seq and ATAC-seq overlapping genes, including one enriched in glycerolipid metabolism (i.e., glycerol kinase, *GK*) and two in the Wnt signaling pathway (i.e., *BAMBI* and *CACYBP*), was qRT-PCR validated, showing different expression patterns during early goose ovarian development. Specifically, the mRNA levels of both *BAMBI* and *CACYBP* increased, but those of *GK* decreased from P4 to P28, indicating their differential actions during the primordial-to-secondary follicle transition.

Taken together, this study represents the first to systematically describe the histomorphological, transcriptomic, and accessible chromatin changes of the goose ovary during late embryonic and early post-hatching stages. Integrated analysis of our ATAC-seq and RNA-seq data demonstrated that chromatin-level regulation of gene activation or repression is achieved via modulation of the accessibility of distinct functional genomic regions that significantly changes in a developmental stage-specific manner. Moreover, this dataset led to the identification of a number of genes, *cis*-regulatory elements, and potential transcriptional factors, which are differentially involved in the regulation of peri-hatching oocyte loss, primordial follicle formation, and their progression to growing follicles. In addition, results from qRT-PCR verified the accuracy of our sequencing data. Therefore, the present study provides a framework for understanding the transcriptome and accessible chromatin dynamics during early avian ovarian development and a new avenue to unravel the transcriptional regulatory mechanisms that facilitate the occurrence of relevant molecular events. Also, this knowledge allows a comparison with the mammalian systems, which may contribute to a comprehensive view of the mechanisms regulating early vertebrate ovarian development and the design of new strategies to manipulate the fertility of humans and domestic animals.

## Data Availability Statement

The datasets generated for this study can be found in the Sequence Read Archive (https://www.ncbi.nlm.nih.gov/sra) at NCBI, with the BioProject ID: PRJNA597548 and SRA Accession Number: SRR10765001-10765020.

## Ethics Statement

The animal study was reviewed and approved by the Institutional Animal Care and Use Committee (IACUC) of Sichuan Agricultural University (Chengdu Campus, Sichuan, China).

## Author Contributions

SH and JW conceptualized and designed this study, reviewed this manuscript, and supervised this study. SH, SY, YL, YD, LiL, JZ, and YZ performed the main experiments and analyzed the data. BH, JH, LX, HH, CH, HL, BK, and LiaL participated in experimental animal management, tissue sampling, and data collection and analysis. SH and SY drafted this manuscript.

## Conflict of Interest

The authors declare that the research was conducted in the absence of any commercial or financial relationships that could be construed as a potential conflict of interest.

## Footnotes

1 https://www.ncbi.nlm.nih.gov/assembly/GCF_000971095.1/

2 http://geneontology.org/

3 https://www.kegg.jp/

## Supplementary Material

FIGURE S1 Periodic acid Schiff (PAS)- and immunohistochemical observations of late embryonic geese ovaries. **(A1,A2)** Photomicrograph of PAS stained ovaries from the E15 and E26 geese embryos, respectively. **(B1,B2)** Photomicrograph of CVH stained ovaries from the E15 and E26 geese embryos, respectively. Arrow indicates the oocyte nests. Scale bar: 50 μm.

FIGURE S2 Principle component analysis (PCA) of the mRNA transcriptome among twelve libraries **(A)** and the ATAC-seq peaks from eight libraries **(B)**. Three and two biological replicates from each of the four representative stages of early ovarian development were subjected to RNA-seq and ATAC-seq, respectively, and respective PCA plot sorted the principle components according to the amounts of data variability.

FIGURE S3 KEGG analyses of nearby genes of differential peaks between different stages of early ovarian development. **(A,B)** Top 20 KEGG pathways of nearby genes of significantly increased and decreased peaks between P0 vs. E15. **(C,D)** Top 20 KEGG pathways of nearby genes of increased and decreased peaks between P4 vs. P0. **(E,F)** Top 20 KEGG pathways of nearby genes of significantly increased and decreased peaks between P28 vs. P4.

FIGURE S4 Genomic views of the ATAC-seq peaks in the nearby of four selected DEGs between different stages of early ovarian development, including *INSR* **(A)**, *TTK* **(B)**, *KL* **(C)**, and *GK* **(D)**. Differential ATAC-seq peaks between different developmental stages are marked with a box.

FIGURE S5 KEGG analyses of nearby DEGs of differential peaks between different stages of early ovarian development. **(A)** Top 20 KEGG pathways of upregulated genes around increased peaks between P0 vs. E15. **(B)** Top 20 KEGG pathways of downregulated genes around decreased peaks between P0 vs. E15. **(C,E)** All KEGG pathways of upregulated genes around increased peaks between P4 vs. P0 and P28 vs. P4, respectively. **(D,F)** All KEGG pathways of downregulated genes around decreased peaks between P4 vs. P0 and P28 vs. P4, respectively.

TABLE S1 Primer pairs for quantitative real-time PCR.

TABLE S2 Quality analysis and mapping of RNA-Seq data to the goose reference genome.

TABLE S3 Overlapping of GO_BP and KEGG terms enriched by the DEGs among three pairwise comparisons.

TABLE S4 Quality analysis and mapping of ATAC-Seq data to the goose reference genome.

## References

[B1] Ackermann A. M. Wang Z. Schug J. Naji A. Kaestner K. H. (2016). Integration of ATAC-seq and RNA-seq identifies human alpha cell and beta cell signature genes. *Mol. Metab.* 5 233–244. 10.1016/j.molmet.2016.01.002 26977395 PMC4770267

[B2] Apostolou E. Hochedlinger K. (2013). Chromatin dynamics during cellular reprogramming. *Nature* 502 462–471. 10.1038/nature12749 24153299 PMC4216318

[B3] Barragan M. Pons J. Ferrer-Vaquer A. Cornet-Bartolome D. Schweitzer A. Hubbard J. (2017). The transcriptome of human oocytes is related to age and ovarian reserve. *Mol. Hum. Reprod.* 23 535–548. 10.1093/molehr/gax033 28586423

[B4] Broekmans F. J. Knauff E. A. te Velde E. R. Macklon N. S. Fauser B. C. (2007). Female reproductive ageing: current knowledge and future trends. *Trends Endocrinol. Metab.* 18 58–65. 10.1016/j.tem.2007.01.004 17275321

[B5] Buenrostro J. D. Giresi P. G. Zaba L. C. Chang H. Y. Greenleaf W. J. (2013). Transposition of native chromatin for fast and sensitive epigenomic profiling of open chromatin, DNA-binding proteins and nucleosome position. *Nat. Methods* 10 1213–1218. 10.1038/nmeth.2688 24097267 PMC3959825

[B6] Buenrostro J. D. Wu B. Chang H. Y. Greenleaf W. J. (2015). ATAC-seq: a method for assaying chromatin accessibility genome-wide. *Curr. Protoc. Mol. Biol.* 109 21 21–29. 10.1002/0471142727.mb2129s109 PMC4374986 25559105

[B7] Cao X. Pfaff S. L. Gage F. H. (2008). YAP regulates neural progenitor cell number via the TEA domain transcription factor. *Genes Dev.* 22 3320–3334. 10.1101/gad.1726608 19015275 PMC2600760

[B8] Carre G. A. Couty I. Hennequet-Antier C. Govoroun M. S. (2011). Gene expression profiling reveals new potential players of gonad differentiation in the chicken embryo. *PLoS ONE* 6:e23959 10.1371/journal.pone.0023959 PMC3170287 21931629

[B9] Collado-Fernandez E. Picton H. M. Dumollard R. (2012). Metabolism throughout follicle and oocyte development in mammals. *Int. J. Dev. Biol.* 56 799–808. 10.1387/ijdb.120140ec 23417402

[B10] Corces M. R. Trevino A. E. Hamilton E. G. Greenside P. G. Sinnott-Armstrong N. A. Vesuna S. (2017). An improved ATAC-seq protocol reduces background and enables interrogation of frozen tissues. *Nat. Methods* 14 959–962. 10.1038/nmeth.4396 28846090 PMC5623106

[B11] Fortune J. E. Cushman R. A. Wahl C. M. Kito S. (2000). The primordial to primary follicle transition. *Mol. Cell. Endocrinol.* 163 53–60. 10.1016/s0303-7207(99)00240-3 10963874

[B12] Ghafari F. Gutierrez C. G. Hartshorne G. M. (2007). Apoptosis in mouse fetal and neonatal oocytes during meiotic prophase one. *BMC Dev. Biol.* 7:87 10.1186/1471-213X-7-87 PMC1965470 17650311

[B13] Gu C. Liu S. Wu Q. Zhang L. Guo F. (2019). Integrative single-cell analysis of transcriptome, DNA methylome and chromatin accessibility in mouse oocytes. *Cell Res.* 29 110–123. 10.1038/s41422-018-0125-4 30560925 PMC6355938

[B14] Guigon C. J. Magre S. (2006). Contribution of germ cells to the differentiation and maturation of the ovary: insights from models of germ cell depletion. *Biol. Reprod.* 74 450–458. 10.1095/biolreprod.105.047134 16339043

[B15] Guioli S. Lovell-Badge R. (2016). RNA FISH, DNA FISH and chromosome painting of chicken oocytes. *Methods Mol. Biol.* 1457 191–208. 10.1007/978-1-4939-3795-0_14 27557582

[B16] Guioli S. Nandi S. Zhao D. Burgess-Shannon J. Lovell-Badge R. Clinton M. (2014). Gonadal asymmetry and sex determination in birds. *Sex Dev.* 8 227–242. 10.1159/000358406 24577119

[B17] Guo H. Hu B. Yan L. Yong J. Wu Y. Gao Y. (2017). DNA methylation and chromatin accessibility profiling of mouse and human fetal germ cells. *Cell Res.* 27 165–183. 10.1038/cr.2016.128 27824029 PMC5339845

[B18] Haba G. Nishigori H. Tezuka Y. Kagami K. Sugiyama T. Nishigori H. (2011). Effect of antithyroid drug on chick embryos during the last week of development: delayed hatching and decreased cerebellar acetylcholinesterase activity. *J. Obstet. Gynaecol. Res.* 37 1549–1556. 10.1111/j.1447-0756.2011.01573.x 21676081

[B19] Hsueh A. J. Kawamura K. Cheng Y. Fauser B. C. (2015). Intraovarian control of early folliculogenesis. *Endocr. Rev.* 36 1–24. 10.1210/er.2014-1020 25202833 PMC4309737

[B20] Hu S. Cao W. Yang M. Liu H. Li L. Wang J. (2014). Molecular characterization, tissue distribution, and expression of two ovarian Dicer isoforms during follicle development in goose (Anser cygnoides). *Compar. Biochem. Physiol. B Biochem. Mol. Biol.* 170 33–41. 10.1016/j.cbpb.2014.01.002 24462910

[B21] Hu S. Liang X. Ren X. Shi Y. Su H. Li Y. (2019). Integrated analysis of mRNA and miRNA expression profiles in the ovary of oryctolagus cuniculus in response to gonadotrophic stimulation. *Front. Endocrinol. (Lausanne)* 10:744 10.3389/fendo.2019.00744 PMC6828822 31736880

[B22] Hunt P. A. Hassold T. J. (2008). Human female meiosis: what makes a good egg go bad? *Trends Genet.* 24 86–93. 10.1016/j.tig.2007.11.010 18192063

[B23] Johnson A. (2011). “Organization and functional dynamics of the avian ovary,” in *Hormones and Reproduction of Vertebrates*, eds Norris D. O. Lopez K. H. (Amsterdam: Elsevier), 71–90. 10.1016/b978-0-12-374932-1.00041-x

[B24] Johnson A. Woods D. C. (2009). Dynamics of avian ovarian follicle development: cellular mechanisms of granulosa cell differentiation. *Gen. Compar. Endocrinol.* 163 12–17. 10.1016/j.ygcen.2008.11.012 19059411

[B25] Kagey M. H. Newman J. J. Bilodeau S. Zhan Y. Orlando D. A. van Berkum N. L. (2010). Mediator and cohesin connect gene expression and chromatin architecture. *Nature* 467 430–435. 10.1038/nature09380 20720539 PMC2953795

[B26] Kang L. Cui X. Zhang Y. Yang C. Jiang Y. (2013). Identification of miRNAs associated with sexual maturity in chicken ovary by Illumina small RNA deep sequencing. *BMC Genomics* 14:352 10.1186/1471-2164-14-352 PMC3700833 23705682

[B27] Kim Y. M. Han J. Y. (2018). The early development of germ cells in chicken. *Int. J. Dev. Biol.* 62 145–152. 10.1387/ijdb.170283jh 29616722

[B28] Krasikova A. Khodyuchenko T. Maslova A. Vasilevskaya E. (2012). Three-dimensional organisation of RNA-processing machinery in avian growing oocyte nucleus. *Chromosome Res.* 20 979–994. 10.1007/s10577-012-9327-7 23318709

[B29] Krysko D. V. Diez-Fraile A. Criel G. Svistunov A. A. Vandenabeele P. D’Herde K. (2008). Life and death of female gametes during oogenesis and folliculogenesis. *Apoptosis* 13 1065–1087. 10.1007/s10495-008-0238-1 18622770

[B30] Levi A. J. Raynault M. F. Bergh P. A. Drews M. R. Miller B. T. Scott R. T. (2001). Reproductive outcome in patients with diminished ovarian reserve. *Fertil. Steril.* 76 666–669. 10.1016/s0015-0282(01)02017-9 11591396

[B31] Li Q. Hu S. Wang Y. Deng Y. Yang S. Hu J. (2019). mRNA and miRNA transcriptome profiling of granulosa and theca layers from geese ovarian follicles reveals the crucial pathways and interaction networks for regulation of follicle selection. *Front. Genet.* 10:988 10.3389/fgene.2019.00988 PMC6820619 31708963

[B32] Liu H. Hu Y. Ji G. Li H. (2014). Rapid-sexing poultries via a new pair of universal primers. *J. Agric. Biotechnol.* 22 1567–1574.

[B33] Liu L. Rajareddy S. Reddy P. Du C. Jagarlamudi K. Shen Y. (2007). Infertility caused by retardation of follicular development in mice with oocyte-specific expression of Foxo3a. *Development* 134 199–209. 10.1242/dev.02667 17164425

[B34] Lowdon R. F. Jang H. S. Wang T. (2016). Evolution of epigenetic regulation in vertebrate genomes. *Trends Genet.* 32 269–283. 10.1016/j.tig.2016.03.001 27080453 PMC4842087

[B35] Mao Z. Fan L. Yu Q. Luo S. Wu X. Tang J. (2018). Abnormality of klotho signaling is involved in polycystic ovary syndrome. *Reprod. Sci.* 25 372–383. 10.1177/1933719117715129 28673204

[B36] Matova N. Cooley L. (2001). Comparative aspects of animal oogenesis. *Dev. Biol.* 231 291–320. 10.1006/dbio.2000.0120 11237461

[B37] Miyamoto K. Nguyen K. T. Allen G. E. Jullien J. Kumar D. Otani T. (2018). Chromatin accessibility impacts transcriptional reprogramming in oocytes. *Cell Rep.* 24 304–311. 10.1016/j.celrep.2018.06.030 29996092 PMC6057489

[B38] Monget P. Bobe J. Gougeon A. Fabre S. Monniaux D. Dalbies-Tran R. (2012). The ovarian reserve in mammals: a functional and evolutionary perspective. *Mol. Cell. Endocrinol.* 356 2–12. 10.1016/j.mce.2011.07.046 21840373

[B39] Nilsson E. E. Savenkova M. I. Schindler R. Zhang B. Schadt E. E. Skinner M. K. (2010). Gene bionetwork analysis of ovarian primordial follicle development. *PLoS ONE* 5:e11637 10.1371/journal.pone.0011637 PMC2905436 20661288

[B40] Oreal E. Mazaud S. Picard J. Y. Magre S. Carre-Eusebe D. (2002). Different patterns of anti-mullerian hormone expression, as related to DMRT1, SF-1, WT1, GATA-4, Wnt-4, and Lhx9 expression, in the chick differentiating gonads. *Dev. Dyn.* 225 221–232. 10.1002/dvdy.10153 12412004

[B41] Pepling M. E. (2006). From primordial germ cell to primordial follicle: mammalian female germ cell development. *Genesis* 44 622–632. 10.1002/dvg.20258 17146778

[B42] Schafer G. L. Ciment G. Stocker K. M. Baizer L. (1993). Analysis of the sequence and embryonic expression of chicken neurofibromin mRNA. *Mol. Chem. Neuropathol.* 18 267–278. 10.1007/bf03160119 8507305

[B43] Schmittgen T. D. Livak K. J. (2008). Analyzing real-time PCR data by the comparative C(T) method. *Nat. Protoc.* 3 1101–1108. 10.1038/nprot.2008.73 18546601

[B44] Smith M. F. Ricke W. A. Bakke L. J. Dow M. P. Smith G. W. (2002). Ovarian tissue remodeling: role of matrix metalloproteinases and their inhibitors. *Mol. Cell. Endocrinol.* 191 45–56. 10.1016/s0303-7207(02)00054-0 12044918

[B45] Stergachis A. B. Neph S. Sandstrom R. Haugen E. Reynolds A. P. Zhang M. (2014). Conservation of trans-acting circuitry during mammalian regulatory evolution. *Nature* 515 365–370. 10.1038/nature13972 25409825 PMC4405208

[B46] Su Y. Q. Sugiura K. Wigglesworth K. O’Brien M. J. Affourtit J. P. Pangas S. A. (2008). Oocyte regulation of metabolic cooperativity between mouse cumulus cells and oocytes: BMP15 and GDF9 control cholesterol biosynthesis in cumulus cells. *Development* 135 111–121. 10.1242/dev.009068 18045843

[B47] Tingen C. Kim A. Woodruff T. K. (2009). The primordial pool of follicles and nest breakdown in mammalian ovaries. *Mol. Hum. Reprod.* 15 795–803. 10.1093/molehr/gap073 19710243 PMC2776475

[B48] Viger R. S. Guittot S. M. Anttonen M. Wilson D. B. Heikinheimo M. (2008). Role of the GATA family of transcription factors in endocrine development, function, and disease. *Mol. Endocrinol.* 22 781–798. 10.1210/me.2007-0513 18174356 PMC2276466

[B49] Wang C. Zhou B. Xia G. (2017). Mechanisms controlling germline cyst breakdown and primordial follicle formation. *Cell Mol. Life Sci.* 74 2547–2566. 10.1007/s00018-017-2480-6 28197668 PMC11107689

[B50] Xu Q. Zhao W. Chen Y. Tong Y. Rong G. Huang Z. (2013). Transcriptome profiling of the goose (Anser cygnoides) ovaries identify laying and broodiness phenotypes. *PLoS ONE* 8:e55496 10.1371/journal.pone.0055496 PMC3566205 23405160

[B51] Yu C. Fan X. Sha Q. Q. Wang H. H. Li B. T. Dai X. X. (2017). CFP1 regulates histone H3K4 trimethylation and developmental potential in mouse oocytes. *Cell Rep.* 20 1161–1172. 10.1016/j.celrep.2017.07.011 28768200

[B52] Yuan X. Xia L. Dong X. Hu S. Zhang Y. Ding F. (2014). Transcription factors GATA-4 and GATA-6: molecular characterization, expression patterns and possible functions during goose (Anser cygnoides) follicle development. *J. Reprod. Dev.* 60 83–91. 10.1262/jrd.2013-080 24531706 PMC3999398

[B53] Zhao D. Lv C. Liu G. Mi Y. Zhang C. (2017). Effect of estrogen on chick primordial follicle development and activation. *Cell Biol. Int.* 41 630–638. 10.1002/cbin.10766 28328180

[B54] Zhao Y. Zhang Y. Li J. Zheng N. Xu X. Yang J. (2018). MAPK3/1 participates in the activation of primordial follicles through mTORC1-KITL signaling. *J. Cell Physiol.* 233 226–237. 10.1002/jcp.25868 28218391

