# Supplementary figures and images for: Dynamics of the Transcriptome and Accessible Chromatin Landscapes During Early Goose Ovarian Development
Source: Front Cell Dev Biol. 2020 Apr 3;8:196. doi: 10.3389/fcell.2020.00196 (PMC7145905; doi:10.3389/fcell.2020.00196)

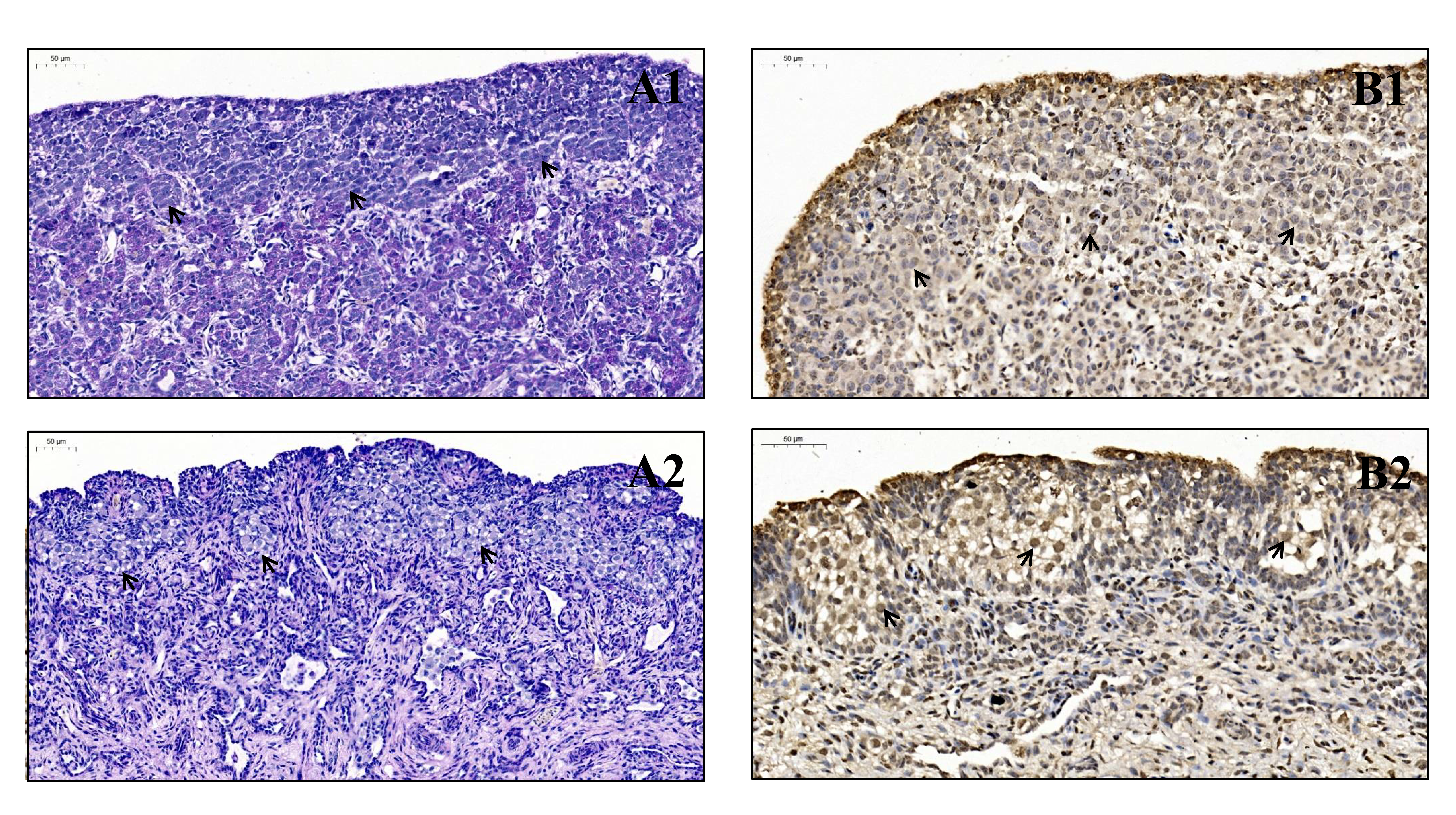

Supplement: FIGURE S1 — Periodic acid Schiff (PAS)- and immunohistochemical observations of late embryonic geese ovaries. (A1,A2) Photomicrograph of PAS stained ovaries from the E15 and E26 geese embryos, respectively. (B1,B2) Photomicrograph of CVH stained ovaries from the E15 and E26 geese embryos, respectively. Arrow indicates the oocyte nests. Scale bar: 50 μm. [file Image_1.TIF]

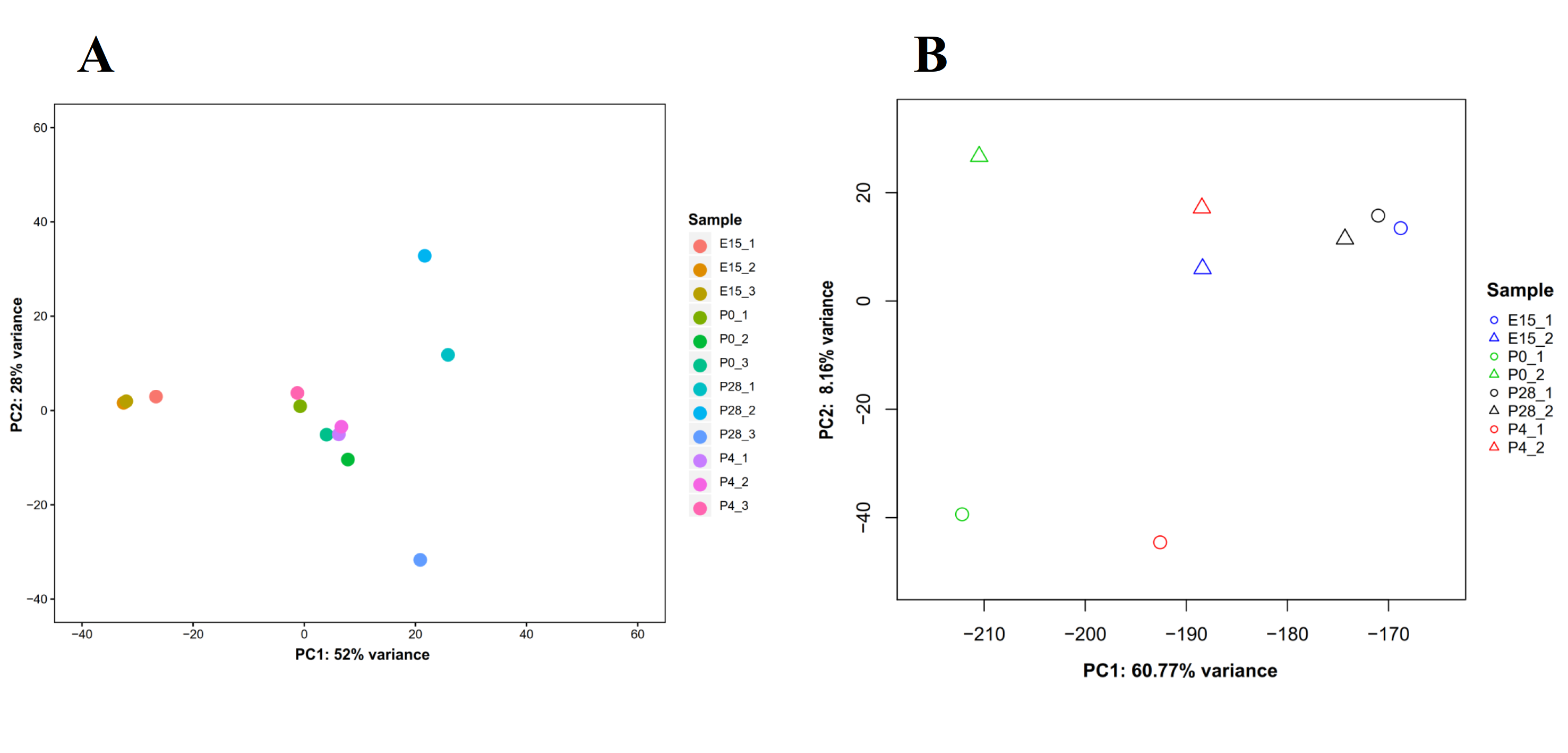

Supplement: FIGURE S2 — Principle component analysis (PCA) of the mRNA transcriptome among twelve libraries (A) and the ATAC-seq peaks from eight libraries (B). Three and two biological replicates from each of the four representative stages of early ovarian development were subjected to RNA-seq and ATAC-seq, respectively, and respective PCA plot sorted the principle components according to the amounts of data variability. [file Image_2.TIF]

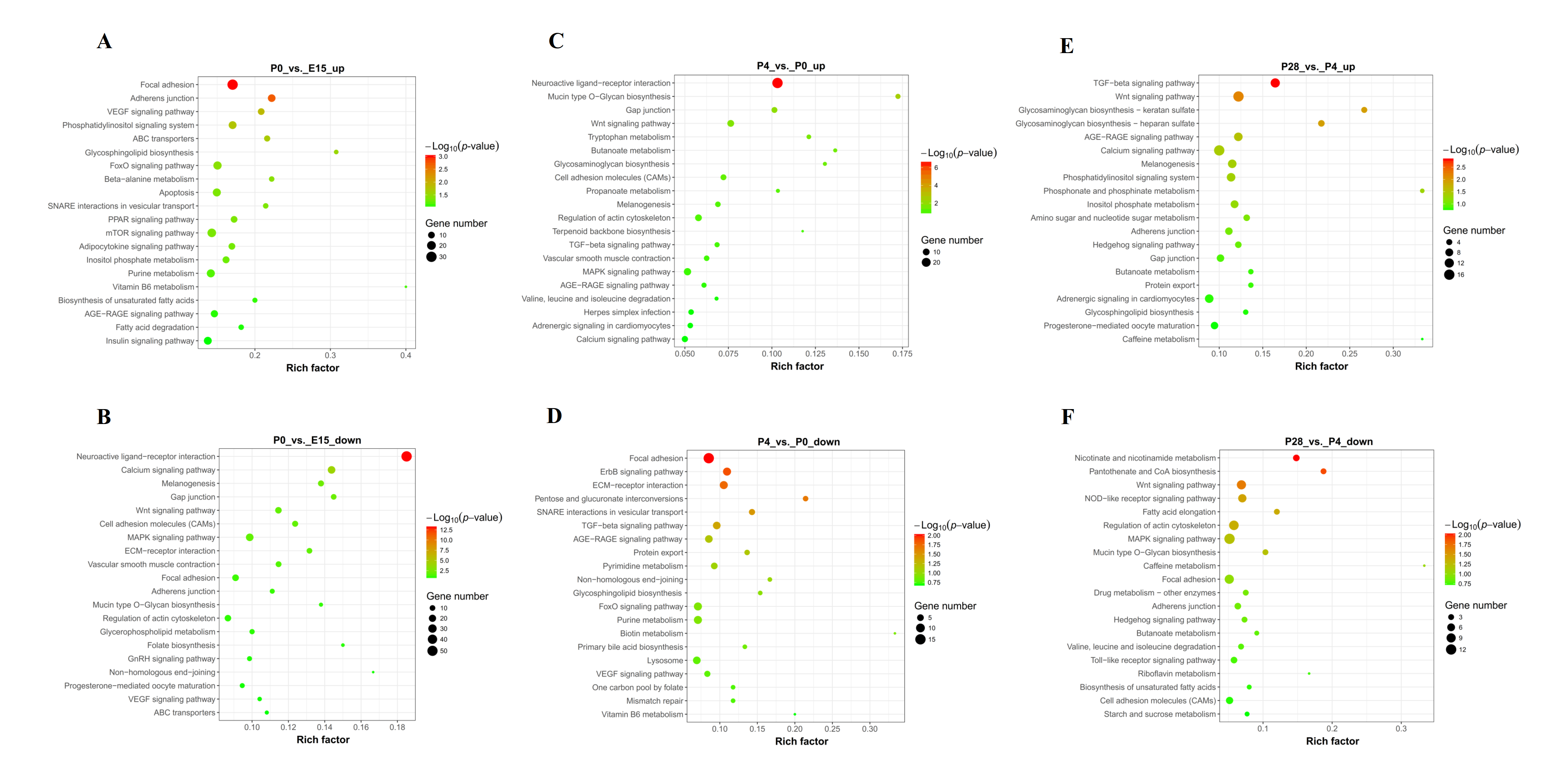

Supplement: FIGURE S3 — KEGG analyses of nearby genes of differential peaks between different stages of early ovarian development. (A,B) Top 20 KEGG pathways of nearby genes of significantly increased and decreased peaks between P0 vs. E15. (C,D) Top 20 KEGG pathways of nearby genes of increased and decreased peaks between P4 vs. P0. (E,F) Top 20 KEGG pathways of nearby genes of significantly increased and decreased peaks between P28 vs. P4. [file Image_3.TIF]

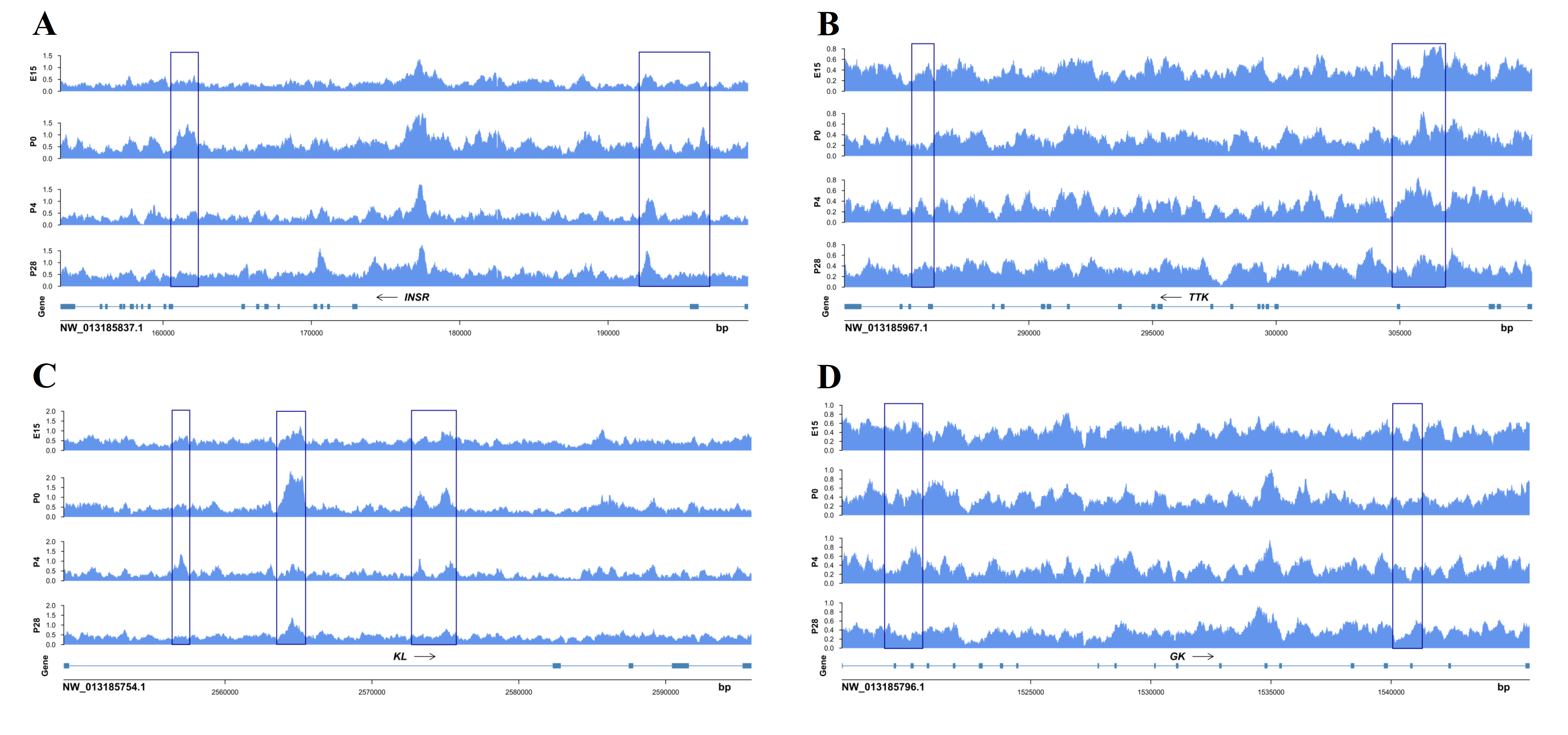

Supplement: FIGURE S4 — Genomic views of the ATAC-seq peaks in the nearby of four selected DEGs between different stages of early ovarian development, including INSR (A), TTK (B), KL (C), and GK (D). Differential ATAC-seq peaks between different developmental stages are marked with a box. [file Image_4.TIF]

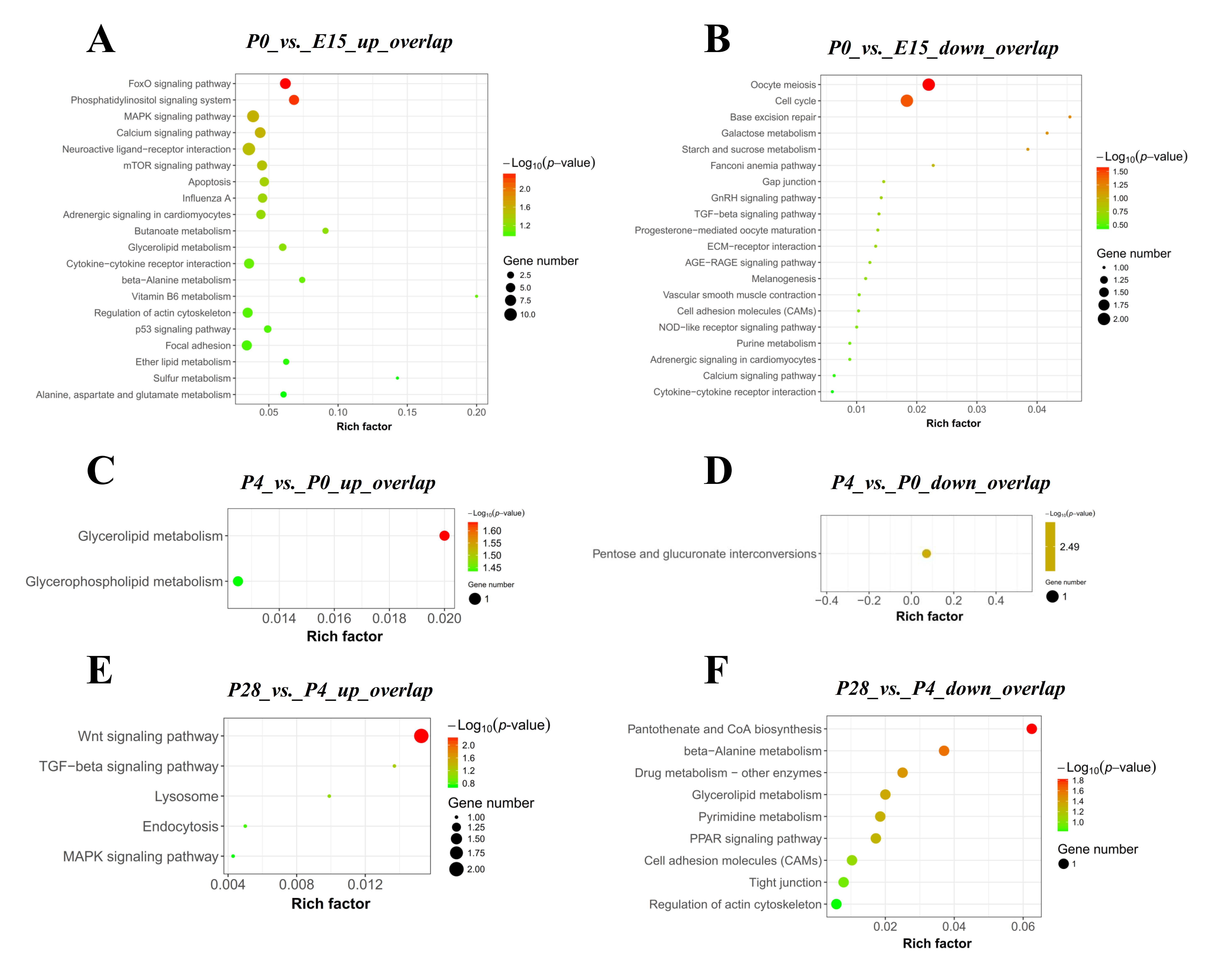

Supplement: FIGURE S5 — KEGG analyses of nearby DEGs of differential peaks between different stages of early ovarian development. (A) Top 20 KEGG pathways of upregulated genes around increased peaks between P0 vs. E15. (B) Top 20 KEGG pathways of downregulated genes around decreased peaks between P0 vs. E15. (C,E) All KEGG pathways of upregulated genes around increased peaks between P4 vs. P0 and P28 vs. P4, respectively. (D,F) All KEGG pathways of downregulated genes around decreased peaks between P4 vs. P0 and P28 vs. P4, respectively. [file Image_5.TIF]
