# Supplementary material for: Dynamics of the Transcriptome and Accessible Chromatin Landscapes During Early Goose Ovarian Development
Source: Front Cell Dev Biol. 2020 Apr 3;8:196. doi: 10.3389/fcell.2020.00196 (PMC7145905; doi:10.3389/fcell.2020.00196)
Supplement: TABLE S1 — Primer pairs for quantitative real-time PCR. [file Table_1.DOCX]

**Suppl. Table 1.** Primer pairs for quantitative real-time PCR

| **Gene symbol** | **Primer sequence (5’to 3’)** | | **Tm (℃)** | **Size (bp)** |
| --- | --- | --- | --- | --- |
| *INSR* | F | GGGAGACAGAGGAAGCACAC | 60.0 | 121 |
|  | R | GCCAGCGATGTAGCTCGTAT |  |  |
| *FASLG* | F | GATCACGCAAAGCACGTTCA | 58.8 | 145 |
|  | R | GTGGGAAGGGAGCAATAGGG |  |  |
| *PI3KR1* | F | ACAAGAAAGAGCGAGAAGAAGACA | 60.0 | 126 |
|  | R | ACCAGCCAATCTCTTCAGGC |  |  |
| *FoxO3* | F | CCGCTTGTCACCGATTTTGG | 60.0 | 119 |
|  | R | ACGGTTTGTTTACCGAGGGG |  |  |
| *PTPN5* | F | ACAGCAGAGTGTGCCTTACC | 60.0 | 92 |
|  | R | TTCCTCTCCTCCATAGCCCC |  |  |
| *DUSP1* | F | AGTATCCCGGTGGAGGACAA | 60.0 | 109 |
|  | R | AGTGCACAAACACCCTTCCT |  |  |
| *MAP3K13* | F | CCAGACTCCAGAGATCCCCA | 60.0 | 193 |
|  | R | TGTGGCATCAGAACACTCCC |  |  |
| *FASN* | F | AGAACCCAGGTGAAACGCTC | 60.0 | 105 |
|  | R | GTGGGAGTGTCATCTCTTCCG |  |  |
| *PRKCA* | F | GGCATGTTTGCTGTTTTGTGG | 59.4 | 189 |
|  | R | TCCCTTGGTGTAGAAGCCCA |  |  |
| *TTK* | F | AATGCAGCCAGATGTGACGA | 60.0 | 161 |
|  | R | TGCATCCCAATGACCACACG |  |  |
| *KL* | F | GACTTCCAGAGCCACGACAA | 60.0 | 104 |
|  | R | GGCAGGTTTTCAGGCAAAGG |  |  |
| *GK* | F | GCCAGATGGAAGAAAGCCGT | 61.4 | 97 |
|  | R | CCAAGGGCAGACTACAGAAGAT |  |  |
| *CACYBP* | F | AACCCATCTCTGTGGAAGGC | 60.0 | 78 |
|  | R | CCTCCCGCTTCTTCCTACAC |  |  |
| *BAMBI* | F | GAGCTTAGCGCCTGCTTTTC | 60.0 | 117 |
|  | R | TGCCTGTTTAGCTTGGCAGA |  |  |
| *ESRRβ* | F | TGTGTACCGCTCCCTACCAT | 60.0 | 200 |
|  | R | TGGAGTCTGAATTGGCGAGG |  |  |
| *GATA2* | F | CGGACTGGCTCACAACTACAT | 60.0 | 110 |
|  | R | CGGAATTGGCGTAGTAGGGG |  |  |
| *GATA3* | F | CTCCTCCACTTTATCCGCCG | 60.0 | 78 |
|  | R | GGGGACACATCTTTGGGAGG |  |  |
| *GATA4* | F | ACAACCACCGAGGAAATGCG | 60.0 | 188 |
|  | R | GGAGACTGGCTGATGGCTGAC |  |  |
| *GATA6* | F | CCAGGAAGCGAAAACCTAAGAAC | 60.0 | 154 |
|  | R | CCTGAAGCTGACGGTTGTGTG |  |  |
| *NF1* | F | CAACTTGCCACTCTCTACTGAA | 58.0 | 155 |
|  | R | GTAAGGGGAGACAATCGCGG |  |  |
| *NR5A2* | F | TGGCTGACCAAACCCTCTTC | 60.0 | 154 |
|  | R | TTCCCATGTACCACTTGCCG |  |  |
| *SMAD3* | F | GTCCTCCATCCTGCCGTTCA | 62.0 | 98 |
|  | R | CCTTCTCGCACCATTTCTCCT |  |  |
| *TEAD1* | F | AGAACAGGGAAGACACGGAC | 58.3 | 98 |
|  | R | GACACCTTAATGGCGGCTTG |  |  |
| *TEAD4* | F | GCCAATATGAGAGCCCGGAA | 60.0 | 150 |
|  | R | CACAAAGAGGGGAACGGTGA |  |  |
| *THRβ* | F | AGGTCCTGCACAGTAACAACA | 59.5 | 196 |
|  | R | TGAGAGTACGATGGCGACTG |  |  |
| *GAPDH* | F | GCTGATGCTCCCATGTTCGTGAT | 60.0 | 86 |
|  | R | GTGGTGCAAGAGGCATTGCTGAC |  |  |
| *β-ACTIN* | F | CAACGAGCGGTTCAGGTGT | 60.0 | 92 |
|  | R | TGGAGTTGAAGGTGGTCTCGT |  |  |

F, forward primer; R, reverse primer
