# Supplementary material for: Dynamics of the Transcriptome and Accessible Chromatin Landscapes During Early Goose Ovarian Development
Source: Front Cell Dev Biol. 2020 Apr 3;8:196. doi: 10.3389/fcell.2020.00196 (PMC7145905; doi:10.3389/fcell.2020.00196)
Supplement: TABLE S2 — Quality analysis and mapping of RNA-Seq data to the goose reference genome. [file Table_2.DOCX]

**Suppl. Table 2.** Quality analysis and mapping of RNA-Seq data to the goose reference genome

| **Sample** | **Number of raw bases**  **(billion)** | **Number of clean bases**  **(billion)** | **Number of raw reads** | **Number of clean reads** | **Q20 ratio (%)** | **Q30 ratio (%)** | **GC content (%)** | **Total mapped genome (%)** | **Unique mapped genome (%)** |
| --- | --- | --- | --- | --- | --- | --- | --- | --- | --- |
| E15_1 | 9.36 | 9.11 | 31211358 | 30363167 | 97.76 | 93.93 | 50.88 | 78.6 | 76.1 |
| E15_2 | 9.04 | 8.79 | 30132675 | 29308619 | 97.55 | 93.46 | 50.94 | 78.2 | 75.8 |
| E15_3 | 8.79 | 8.55 | 29304870 | 28495393 | 97.46 | 93.27 | 51.23 | 77.5 | 75.2 |
| P0_1 | 9.03 | 8.80 | 30114344 | 29347817 | 98.00 | 94.62 | 50.32 | 79.4 | 75.5 |
| P0_2 | 8.59 | 8.41 | 28640067 | 28048645 | 97.79 | 93.93 | 51.23 | 79.9 | 77.4 |
| P0_3 | 8.62 | 8.44 | 28739870 | 28126539 | 97.74 | 93.84 | 50.64 | 80.4 | 77.6 |
| P4_1 | 8.45 | 8.23 | 28163344 | 27444111 | 97.74 | 93.85 | 50.26 | 80.8 | 76.9 |
| P4_2 | 8.46 | 8.24 | 28211224 | 27458527 | 97.81 | 94.01 | 51.01 | 80.1 | 76.9 |
| P4_3 | 9.40 | 9.20 | 31343766 | 30666675 | 97.71 | 93.77 | 50.52 | 80.3 | 77.7 |
| P28_1 | 8.68 | 8.49 | 28931047 | 28295734 | 97.77 | 93.89 | 50.63 | 81.1 | 78.5 |
| P28_2 | 8.47 | 8.27 | 28237467 | 27579847 | 97.70 | 93.71 | 50.64 | 81.4 | 79.3 |
| P28_3 | 12.06 | 11.78 | 40211959 | 39268196 | 97.69 | 93.70 | 51.15 | 80.7 | 79.3 |
