# Supplementary material for: Dynamics of the Transcriptome and Accessible Chromatin Landscapes During Early Goose Ovarian Development
Source: Front Cell Dev Biol. 2020 Apr 3;8:196. doi: 10.3389/fcell.2020.00196 (PMC7145905; doi:10.3389/fcell.2020.00196)
Supplement: TABLE S3 — Overlapping of GO_BP and KEGG terms enriched by the DEGs among three pairwise comparisons. [file Table_3.DOCX]

**Suppl. Table 3.** Overlapping of GO_BP and KEGG terms enriched by the DEGs among three pairwise comparisons

| **Overlap** | **GO ID** | **GO_BP Term** | **Commonly involved DEGs** | **KEGG ID** | **KEGG Pathway Term** | **Commonly involved DEGs** |
| --- | --- | --- | --- | --- | --- | --- |
| (P0*vs.*E15)∩(P4*vs.*P0) | GO:0045494 | Photoreceptor cell maintenance | *CDHR1, RP1L1* | PATH:04114 | Oocyte meiosis | *SGOL1* |
| (P0*vs.*E15)∩(P28*vs.*P4) | GO:0055085 | Transmembrane transport | *SLC22A7, SLC2A5,*  *SLC5A10, SLC16A4* | PATH:04512 | ECM-receptor interaction | *THBS2, TNC,*  *COL6A6, TNN,*  *SPP1, VTN* |
|  | GO:0006836 | Neurotransmitter transport | *SLC6A19* | PATH:04310 | Wnt signaling pathway | *WNT11, CACYBP,*  *MMP7, BAMBI, LGR5* |
|  | GO:0006807 | Nitrogen compound metabolic process | *UPB1, LOC106032503* | PATH:00260 | Glycine, serine and threonine metabolism | *DAO, CTH,*  *AGXT, GAMT, PIPOX* |
|  |  |  |  | PATH:00330 | Arginine and proline metabolism | *DAO, GAMT, L3HYPDH, CKMT2* |
|  |  |  |  | PATH:03320 | PPAR signaling pathway | *ACSL6* |
|  |  |  |  | PATH:00380 | Tryptophan metabolism | *HAAO* |
|  |  |  |  | PATH:04510 | Focal adhesion | *THBS2, TNC, COL6A6, TNN, SPP1, VTN* |
| (P4*vs.*P0)∩(P28*vs.*P4) | GO:0019441 | Tryptophan catabolic process to kynurenine | *IDO2* | PATH:04514 | Cell adhesion molecules (CAMs) | *PTPRC, CD2* |
|  | GO:0021819 | Layer formation in cerebral cortex | *LRP8* |  |  |  |
|  | GO:0050829 | Defense response to Gram-negative bacterium | *MMP7* |  |  |  |
