# Supplementary material for: Dynamics of the Transcriptome and Accessible Chromatin Landscapes During Early Goose Ovarian Development
Source: Front Cell Dev Biol. 2020 Apr 3;8:196. doi: 10.3389/fcell.2020.00196 (PMC7145905; doi:10.3389/fcell.2020.00196)
Supplement: TABLE S4 — Quality analysis and mapping of ATAC-Seq data to the goose reference genome. [file Table_4.DOCX]

**Suppl. Table 4.** Quality analysis and mapping of ATAC-Seq data to the goose reference genome

| **Sample** | **Number of raw bases**  **(billion)** | **Number of clean bases**  **(billion)** | **Number of raw reads** | **Number of clean reads** | **Q20 ratio (%)** | **Q30 ratio (%)** | **GC content (%)** | **Total mapped genome (%)** | **Unique mapped genome (%)** |
| --- | --- | --- | --- | --- | --- | --- | --- | --- | --- |
| E15_1 | 21.51 | 15.82 | 71689523 | 71290779 | 91.86 | 85.19 | 45.03 | 92.5 | 87.6 |
| E15_2 | 14.08 | 11.07 | 46931789 | 46694251 | 92.77 | 86.03 | 46.45 | 92.7 | 86 |
| P0_1 | 17.91 | 13.03 | 59699924 | 59335721 | 91.62 | 84.59 | 47.37 | 91.3 | 86.1 |
| P0_2 | 17.28 | 13.07 | 57605977 | 57296103 | 92.14 | 85.34 | 46.78 | 92.2 | 86 |
| P4_1 | 14.62 | 11.51 | 48739506 | 48543361 | 93.49 | 87.36 | 47.12 | 94.3 | 87.1 |
| P4_2 | 14.11 | 11.15 | 47023830 | 46852327 | 93.69 | 87.66 | 47.51 | 94.1 | 86.5 |
| P28_1 | 36.33 | 14.92 | 121093247 | 66669433 | 92.38 | 85.86 | 46.53 | 89.7 | 83.8 |
| P28_2 | 22.21 | 16.43 | 74024120 | 73635500 | 91.87 | 84.95 | 47.42 | 92.8 | 85 |
